# Dietary fat and fatty acid consumptions and the odds of asthenozoospermia: a case–control study in China

**DOI:** 10.1093/hropen/hoad030

**Published:** 2023-07-27

**Authors:** Jun-Qi Zhao, Xiao-Bin Wang, Xu Leng, Yi-Fan Wei, Dong-Hui Huang, Jia-Le Lv, Qiang Du, Ren-Hao Guo, Bo-Chen Pan, Qi-Jun Wu, Yu-Hong Zhao

**Affiliations:** Department of Clinical Epidemiology, Shengjing Hospital of China Medical University, Shenyang, China; Clinical Research Center, Shengjing Hospital of China Medical University, Shenyang, China; Center of Reproductive Medicine, Shengjing Hospital of China Medical University, Shenyang, China; Center of Reproductive Medicine, Shengjing Hospital of China Medical University, Shenyang, China; Department of Clinical Epidemiology, Shengjing Hospital of China Medical University, Shenyang, China; Clinical Research Center, Shengjing Hospital of China Medical University, Shenyang, China; Department of Clinical Epidemiology, Shengjing Hospital of China Medical University, Shenyang, China; Clinical Research Center, Shengjing Hospital of China Medical University, Shenyang, China; Department of Clinical Epidemiology, Shengjing Hospital of China Medical University, Shenyang, China; Clinical Research Center, Shengjing Hospital of China Medical University, Shenyang, China; Center of Reproductive Medicine, Shengjing Hospital of China Medical University, Shenyang, China; Center of Reproductive Medicine, Shengjing Hospital of China Medical University, Shenyang, China; Center of Reproductive Medicine, Shengjing Hospital of China Medical University, Shenyang, China; Department of Clinical Epidemiology, Shengjing Hospital of China Medical University, Shenyang, China; Clinical Research Center, Shengjing Hospital of China Medical University, Shenyang, China; Department of Obstetrics and Gynecology, Shengjing Hospital of China Medical University, Shenyang, China; NHC Key Laboratory of Advanced Reproductive Medicine and Fertility (China Medical University), National Health Commission, Shenyang, China; Department of Clinical Epidemiology, Shengjing Hospital of China Medical University, Shenyang, China; Clinical Research Center, Shengjing Hospital of China Medical University, Shenyang, China

**Keywords:** asthenozoospermia, case–control study, China, diet, fat, fatty acid

## Abstract

**STUDY QUESTION:**

Are dietary fat and fatty acid (FA) intakes related to the odds of asthenozoospermia?

**SUMMARY ANSWER:**

Plant-based fat consumption was associated with decreased asthenozoospermia odds, while the consumption of animal-based monounsaturated fatty acid (MUFA) was positively related to asthenozoospermia odds.

**WHAT IS KNOWN ALREADY:**

Dietary fat and FA are significant ingredients of a daily diet, which have been demonstrated to be correlated to the reproductive health of men. However, to date, evidence on fat and FA associations with the odds of asthenozoospermia is unclear.

**STUDY DESIGN, SIZE, DURATION:**

The hospital-based case–control study was performed in an infertility clinic from June 2020 to December 2020. Briefly, 549 asthenozoospermia cases and 581 controls with normozoospermia were available for final analyses.

**PARTICIPANTS/MATERIALS, SETTING, METHODS:**

We collected dietary data through a verified food frequency questionnaire of 110 food items. Asthenozoospermia cases were ascertained according to the World Health Organization guidelines. To investigate the correlations of dietary fat and FA consumptions with the odds of asthenozoospermia, we calculated the odds ratios (ORs) and corresponding 95% CIs through unconditional logistic regression models.

**MAIN RESULTS AND THE ROLE OF CHANCE:**

Relative to the lowest tertile of consumption, the highest tertile of plant-based fat intake was inversely correlated to the odds of asthenozoospermia (OR = 0.68, 95% CI = 0.50–0.91), with a significant dose–response relation (OR = 0.85, 95% CI = 0.75–0.97, per standard deviation increment). Inversely, animal-based MUFA intake (OR = 1.49, 95% CI = 1.04–2.14) was significantly correlated to increased odds of asthenozoospermia, and an evident dose–response relation was also detected (OR = 1.24, 95% CI = 1.05-1.45, per standard deviation increment). Subgroup analyses showed similar patterns of associations to those of the primary results. Moreover, we observed significant interactions on both multiplicative and additive scales between animal-based MUFA and cigarette smoking.

**LIMITATIONS, REASONS FOR CAUTION:**

Selection bias and recall bias were unavoidable in any of the observational studies. As we failed to obtain the information of trans-fatty acid (TFA) consumption, the relation of TFA intake and asthenozoospermia odds was unclear.

**WIDER IMPLICATIONS OF THE FINDINGS:**

This study indicated that different sources of fat and FAs might exert different effects on the etiology of asthenozoospermia, and cigarette smoking could exacerbate the adverse effect of high animal-based MUFA intake on asthenozoospermia. Our findings provide novel evidence pertaining to the fields of prevention of asthenozoospermia through decreasing animal-derived fat and FA consumptions and smoking cessation.

**STUDY FUNDING/COMPETING INTEREST(S):**

This work was supported by the JieBangGuaShuai Project of Liaoning Province, Natural Science Foundation of Liaoning Province, Clinical Research Cultivation Project of Shengjing Hospital, and Outstanding Scientific Fund of Shengjing Hospital. All authors have no conflict of interest to declare.

**TRIAL REGISTRATION NUMBER:**

N/A.

WHAT DOES THIS MEAN FOR PATIENTS? As a common cause of male infertility, asthenozoospermia (a condition in which a person has decreased sperm motility) is involved in >80% male infertility cases. Recent evidence has shown that many factors, including unhealthy lifestyle, environmental pollutants, infections, and genetic factors, are related to asthenozoospermia. However, these aforementioned factors are hard to change and the identification of factors that could be altered, for example by varying the diet, is likely to be significant for the prevention of asthenozoospermia. Fat and fatty acids are the main components of a daily diet and play a crucial role in multiple health outcomes. Recent studies suggest that fat and fatty acids are associated with sperm quality and the chance (odds) of developing asthenozoospermia. However, current evidence for a relation of dietary fat and fatty acid intakes with asthenozoospermia is lacking. Hence, we carried out a study with 549 asthenozoospermia cases and 581 controls with normal sperm to investigate this topic more thoroughly. We found that plant-based fat consumption was related to decreased asthenozoospermia odds, whereas the consumption of animal-based monounsaturated fatty acid was linked to increased odds of asthenozoospermia. Among the common fatty acids, palmitoleic acid, stearic acid, and arachidonic acid, which commonly exist in red meat, ultra-processed foods, and animal oil, were correlated to increased odds of asthenozoospermia. Further analyses suggested that cigarette smoking might increase the effect of animal-based monounsaturated fatty acid on increasing the odds of asthenozoospermia. Further studies are underway to identify which dietary factors could be modified to minimize the risk of infertility.

## Introduction

The global burden of infertility is estimated at 48 million couples (Boivin *et al.*, 2007), affecting ∼15% of reproductive-age couples, of which >50% of cases are attributed to male factors (Patel *et al.*, 2018). With the continuing decline of sperm quality, the rate of male infertility may be rising (Bonde *et al.*, 1998; Skakkebæk *et al.*, 2022; Levine *et al.*, 2023). As a common cause of male infertility, asthenozoospermia is involved in >80% of primary male infertility cases (Curi *et al.*, 2003), which is defined as the proportion of total motile or progressive motility spermatozoa below the lower reference values of the World Health Organization (WHO) guidelines (World Health Organization, 2010; Eslamian *et al.*, 2020). Recent evidence indicated that multifarious factors, including unhealthy lifestyle (Adams *et al.*, 2014; Jóźków *et al.*, 2017), environmental pollutants (Benoff *et al.*, 2008; Martini *et al.*, 2010), varicocele (Kurtz *et al.*, 2015), infections (Cai *et al.*, 2014), and genetic factors (Zuccarello *et al.*, 2008), were related to asthenozoospermia. Compared with these aforementioned factors, diet is more modifiable for preventative interventions. Moreover, the shift from unprocessed agricultural diets to modern processed diets has not only significantly impaired reproductive health but also could contribute to a gradual decline in the reproductive function of subsequent generations (Whittaker, 2023).

Fat and fatty acids (FAs) are main components of a daily diet, which not only enhance the organoleptic properties of food through improving the taste, texture, flavor, and aroma, thus affecting the acceptability and palatability of foods, but also provide significant fat-soluble vitamins (A, D, E, K) and phytochemicals to the human body (Uauy and Dangour, 2009; Zhao *et al.*, 2022). Accumulated evidence has indicated that dietary fat and FAs play a crucial role in visual and cognitive development (Uauy *et al.*, 2001), cardiovascular health (Calder, 2015), immune function (Radzikowska *et al.*, 2019), and reproductive health (Eslamian *et al.*, 2015). Previous research also suggested that omega-3 polyunsaturated fatty acid (PUFA), an important component of sperm cell membranes, could affect the ability of sperm to stimulate hormone production and fertilize an egg (Eslamian *et al.*, 2015). Moreover, a case–control study with 107 asthenozoospermia cases and 235 age-matched controls demonstrated that trans-fatty acid (TFA) and saturated fatty acid (SFA) intakes were positively correlated to the odds of developing asthenozoospermia, while inverse relations were found between omega-3 PUFA and the odds of asthenozoospermia (Eslamian *et al.*, 2015). Hence, dietary fat and FA consumptions might exert a crucial impact on sperm quality, especially in the etiology of asthenozoospermia.

Notwithstanding, as the sample size was relatively small and different food sources were not taken into account, existing evidence on the relations of dietary FA consumptions with the odds of asthenozoospermia is inconclusive (Eslamian *et al.*, 2015). Meanwhile, no research has investigated the association of dietary fat with the odds of asthenozoospermia. Consequently, we performed this case–control study to make a thorough inquiry about fat and FA consumptions and the odds of asthenozoospermia in the Chinese population and provide some enlightenment for further studies.

## Materials and methods

### Study design and population

**Figure 1.**
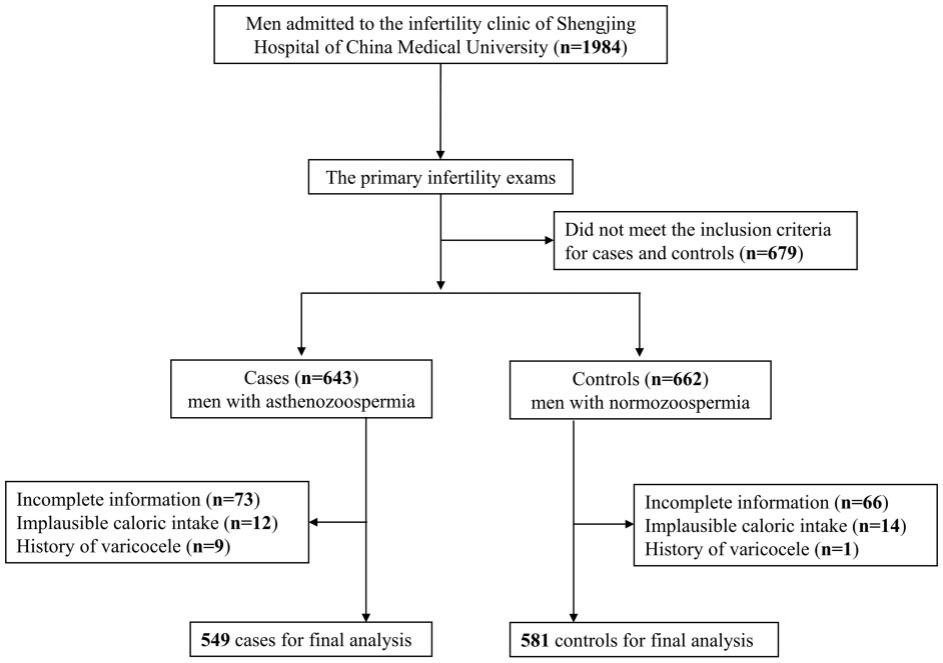
**Flow diagram of the selection of men for the hospital-based case–control study to assess the relations of dietary fat and fatty acid consumptions to asthenozoospermia**.

In this case–control study, participants who referred to the infertility clinic of Shengjing Hospital of China Medical University between June 2020 and December 2020 were enrolled. Overall, 1984 men were available for the current study. Participants were divided into two groups after the primary infertility exams, in accordance with the WHO laboratory manual for the examination and processing of human semen (World Health Organization, 2010). Asthenozoospermia cases (n = 643) were defined as the percentage of total motility (including progressive motility and non-progressive motility) <40% or progressive motility (including slowly and rapidly progressive motility) <32% according to WHO guidelines (Efrat *et al.*, 2018). Normozoospermia men from the same infertility clinic (n = 662) were assigned to the control group. A baseline questionnaire, including dietary and sociodemographic information, was administered by well-trained interviewers to all recruited participants. Participants with extreme total caloric intake (<800 or >4200 kcal/day) (n = 26), incomplete information (n = 139), or a history of varicocele (n = 10) were further excluded (Eslamian *et al.*, 2012, 2015; Liu *et al.*, 2021; Lv *et al.*, 2022). After the exclusion of ineligible participants, 549 cases and 581 controls were applicable for statistical analysis (Fig. 1). The ethical protocol was authorized by the Ethics Committee of Shengjing Hospital of China Medical University. All participants signed informed consent forms before participation.

### Semen collection and analysis

After a 3 to 7 day period of abstinence, participants were asked to collect a semen sample through masturbation into a sterilized tube. Lubricants and condoms were forbidden in this procedure. Before analyses, all semen samples were liquefied for at least 45 minutes. The volume of ejaculate was directly measured, and the pH of semen was assessed with a standard pH test strip. Sperm morphology was observed through a 1000× oil microscope, and the percentage of normal sperm morphology was determined by counting 200 intact sperm.

Total motility, the percentage of each motile grade of sperm, total sperm count, and sperm concentration were examined with WLJY9000 (Beijing Weili New Century Science & Tech. Dev. Co. Ltd. Beijing, China), a computer-aided sperm analysis system. The percentage of motile sperm was defined according to the WHO laboratory manual for the examination and processing of human semen: velocity >25 μm/s at 37°C was defined as grade A; velocity at 37°C > 5 μm/s and <25 μm/s was defined as grade B; grade C was defined as velocity <5 μm/s at 37°C; sperm that did not move at 37°C were defined as grade D. Each semen sample was examined twice by two experienced technicians, and the reference values of normal sperm were identified according to the WHO criteria (World Health Organization, 2021).

External quality was governed by trained technicians. This programme was undertaken by joining a national quality control program for semen analysis, which was arranged by the Society of Reproductive Medicine, Chinese Medical Association (Li *et al.*, 2022). Four technicians detected the control semen samples from the Central Lab for total sperm count, total motility, sperm morphology, and sperm concentration, and the average values were sent back for evaluation and monitoring. This procedure could contribute to detect deviations and assure quality.

### Data collection

Baseline characteristics, including age, cigarette smoking, alcohol drinking, dietary change, education, physical activity, and annual household income, were gathered using a structured questionnaire. Cigarette smoking and alcohol drinking were defined as participants who smoked or drank at least 1 time/day or 1 time/week, for >6 consecutive months. Dietary change was defined as participants who had made any changes to their dietary habits recently with four appropriate responses: from this year, from 1 to 2 years ago, from 3 years ago, and none. Weight and height were estimated with a standard protocol, and BMI was obtained through weight (kg)/height (m^2^). All participants were asked to report the usual type and duration of physical activities in relation to work, exercise, housework, and commuting over the past year (Du *et al.*, 2013; Zhao *et al.*, 2023). After that, the metabolic equivalent (MET) of each activity was estimated through multiplying the frequency by the duration, subsequently summing up each activity to calculate total physical activity in MET hours per week (Ainsworth *et al.*, 2011; Du *et al.*, 2013).

### Dietary assessment

Dietary information was measured via a verified 110-item food frequency questionnaire (FFQ) at baseline, which was performed by experienced and well-trained personnel. The FFQ was designed to evaluate the frequency of dietary intake and supplement use over the past year before admission to the infertility clinic (Cui *et al.*, 2022b; Huang *et al.*, 2023), and its validity and reliability have been proved by our previous research (Liu *et al.*, 2021; Wang *et al.*, 2021a; Cui *et al.*, 2022a). The reproducibility coefficients were above 0.5 for most food groups, and Spearman correlation coefficients ranged from 0.3 to 0.7 for most food groups between weighed diet records and the FFQ (Cui *et al.*, 2022b; Liu *et al.*, 2022). Seven response options of usual consumption frequency of each food (i.e. >2 times/day, 1–2 times/day, 4–6 times/week, 2–3 times/week, 1 time/week, 2–3 times/month, and almost never) were available for participants to choose. Each food consumption was obtained by multiplying the frequency with the fitted portion sizes (gram/time) (Zhang *et al.*, 2021). Nutrient intakes were estimated via linking the Chinese food composition table to the dietary data (Hu *et al.*, 2018; Yang *et al.*, 2018).

Dietary fat was separated into animal-based fat and plant-based fat in line with food sources. Total FA intake includes SFA, monounsaturated fatty acid (MUFA), and PUFA. SFA was further divided into short-to-medium-chain SFA [saturated butyric (C4), caproic (C6), caprylic (C8), capric (C10), undecanoic (C11), lauric (C12), and tridecanoic (C13) acids] and long-chain SFA [saturated myristic (C14), pentadecanoic (C15), palmitic (C16), heptadecanoic (C17), stearic (C18), nonadecanoic (C19), arachidic (C20), behenic (C22), and lignoceric (C24) acids] based on the length of the carbon chain (Yang *et al.*, 2018). MUFA was similarly divided into animal-based MUFA and plant-based MUFA according to the food sources. PUFA was further separated into omega-3 PUFA [docosapentaenoic acid (DPA), eicosapentaenoic acid (EPA), and docosahexaenoic acid (DHA), parinaric acid, alpha-linolenic acid, and docosatrienoic acid] and omega-6 PUFA (linoleic acid, eicosadienoic acid, arachidonic acid, and docosatetraenoic acid) based on the position of the double bond. Furthermore, we calculated the consumption of marine (i.e. mainly of fish origin) omega-3 PUFA (EPA, DPA, and DHA) and the ratio of omega-6 PUFA and omega-3 PUFA.

### Statistical analysis

We performed the Kolmogorov–Smirnov test to assess the normality of continuous variables. Differences in dietary, sociodemographic, and sperm quality characteristics between the two groups were examined using the Chi-square test for categorical variables, and continuous variables were examined using the Kruskal–Wallis test as none of them fit the normal distribution. Values were displayed as number with percentage for categorical variables and median with interquartile range (IQR) for continuous variables. Dietary fat and FA consumptions were categorized into tertiles according to the consumptions of controls, and the lowest tertile was considered as the reference category. All nutrients were adjusted for total energy intake with the residual method in the present analysis (Willett and Stampfer, 1986). We used unconditional logistic regression to estimate the odds ratios (ORs) and corresponding 95% CIs for the associations between dietary fat and FA consumptions and the odds of asthenozoospermia. We also investigated the associations of several common FA consumptions with the odds of asthenozoospermia. *P* values for linear trend were calculated by assigning the median value of each tertile as a continuous variable in logistic regression models. Furthermore, the nonlinear relations between dietary fat and FA intakes and asthenozoospermia odds were tested through the penalized cubic splines with three equally spaced knots (i.e. 5, 50, and 95th percentiles) (Govindarajulu *et al.*, 2009).

We constructed three models to evaluate the relations, and confounders selection was determined by the association with clinical features, dietary fat and FA consumptions, and previous research (Eslamian *et al.*, 2015; Wang *et al.*, 2021b; Cui *et al.*, 2022a). Specifically, we adjusted for total energy intake (kcal/day) and age (years) in Model 1. Model 2 was further adjusted for abstinence time (days), BMI (kg/m^2^), physical activity (MET/hours/week), dietary change (yes/no), cigarette smoking (yes/no), alcohol drinking (yes/no), annual household income (<50, 50 to <100, or ≥100 thousand yuan), and education (junior secondary or below, senior high school/technical secondary school, and junior college/university or above). Total protein (g/day) and total carbohydrate intake (g/day) were further adjusted in Model 3.

Subgroup analyses were performed to assess the effect of modifications according to cigarette smoking (yes versus no), alcohol drinking (yes versus no), BMI (<25 versus ≥25 kg/m^2^), age (<32 versus ≥32 years), and physical activity (≤127.57 versus >127.57 MET/hours/week), which are potential risk factors of asthenozoospermia. Physical activity was divided into two categories according to the median value of the control group. The *P* value for multiplicative interactions was determined by the likelihood-ratio test for the product terms between dietary fat and FA consumptions and these stratified variables. Besides, we further estimated relative excess risk due to the additive interactions between fat and FA consumptions and these stratified variables to ensure the robustness of the interactions. We also conducted several sensitivity analyses to assess the robustness of the primary results. We first adjusted for total energy intake using the nutrient density method to evaluate the effect of different energy-adjusted methods on these associations. Besides, we excluded the participants who had ever changed their dietary habits to alleviate the concern for the impact of dietary change on the associations. We used SAS software, version 9.4 (SAS Institute, Cary, NC, USA), for all statistical analyses. All statistical tests were two sided, with a *P* value of <0.05 considered to be significant.

## Results

### Baseline characteristics of the study population

Table 1 shows the distribution of baseline characteristics in the asthenozoospermia cases and normal controls. Relative to the control group, asthenozoospermia cases were slightly older, had a lower proportion of alcohol drinking, and experienced a longer period of abstinence times (all *P *<* *0.05). With regard to semen parameters, asthenozoospermia cases had lower total sperm count, sperm concentration, total motility, progress motility, and percentage of normal sperm morphology (all *P *<* *0.05) than controls. Moreover, asthenozoospermia cases consumed relatively more carbohydrate, long-chain SFA, and animal-based MUFA, but relatively less plant-based fat (all *P *<* *0.05).

**Table 1.** General characteristics of asthenozoospermia cases and normal controls.

Characteristics	Cases	Controls	*P* value*
**No. of participants**	549	581	
**Age (years), median (IQR)**	33.00 (30.00–36.00)	32.00 (29.00–34.00)	<0.05
**BMI (kg/m^2^), median (IQR)**	26.17 (23.72–28.73)	25.95 (23.36–28.73)	0.36
**Physical activity (MET/hours/week), median (IQR)**	132.83 (100.70–217.17)	127.57 (98.35–226.67)	0.67
**Ever smoking (n, %)**	264 (48.09)	307 (52.84)	0.11
**Ever alcohol drinking (n, %)**	199 (36.25)	250 (43.03)	<0.05
**Ever dietary change (n, %)**	127 (23.13)	115 (19.79)	0.17
**Educational level (n, %)**			0.67
Junior secondary or below	121 (22.04)	141 (24.27)	
Senior high school/technical secondary school	79 (14.39)	82 (14.11)	
Junior college/university or above	349 (63.57)	358 (61.62)	
**Annual family income (thousand yuan), (n, %)**			0.76
<50	98 (17.85)	94 (16.18)	
50 to <100	209 (38.07)	226 (38.90)	
≥100	242 (44.08)	261 (44.92)	
**Abstinence time (days), median (IQR)**	4.00 (3.00–5.00)	4.00 (3.00–5.00)	<0.05**
**Semen parameters, median (IQR)**			
Ejaculate volume (ml)	3.40 (2.50–4.40)	3.20 (2.50–4.00)	0.12
Sperm concentration (10^6^/ml)	49.13 (33.01–73.44)	62.26 (42.63–87.59)	<0.05
Total sperm count (10^6^/ml)	169.20 (108.40–255.42)	211.18 (131.00–297.53)	<0.05
Progressive motility (%)	23.18 (15.93–28.41)	43.06 (37.85–50.40)	<0.05
Total motility (%)	29.06 (20.77–35.38)	53.65 (46.44–62.23)	<0.05
Normal sperm morphology (%)	5.00 (4.00–7.00)	6.00 (4.00–8.00)	<0.05
**Diet, median (IQR)** ^†^			
Total energy intake (kcal/day)	1731.23 (1401.51–2149.24)	1683.75 (1400.51–2048.60)	0.12
Total protein intake (g/day)	73.96 (68.47–79.56)	73.91 (68.62–79.37)	0.92
Total carbohydrate intake (g/day)	257.71 (236.81–277.03)	254.63 (233.61–271.78)	<0.05
Total fiber intake (g/day)	16.81 (14.23–19.58)	16.46 (13.75–19.51)	0.17
Cholesterol intake (g/day)	370.81 (277.69–485.86)	361.15 (279.77–469.41)	0.36
Total fat intake (g/day)	50.27 (44.31–55.83)	49.47 (45.11–55.65)	0.96
Animal-based fat intake (g/day)	26.72 (22.30–31.87)	27.45 (22.62–32.66)	0.10
Plant-based fat intake (g/day)	21.75 (18.55–26.15)	23.01 (18.97–26.86)	<0.05
Total FA intake (g/day)	37.86 (33.03–42.91)	38.71 (34.03–43.37)	0.23
Total SFA intake (g/day)	15.05 (12.78–17.31)	15.37 (13.34–17.47)	0.08
Short-to-medium-chain SFA^a^ intake (g/day)	0.71 (0.47–0.97)	0.67 (0.48–0.91)	0.30
Long-chain SFA^b^ intake (g/day)	15.32 (13.25–17.22)	14.91 (12.68–16.94)	<0.05
Total MUFA intake (g/day)	15.45 (13.32–17.53)	15.66 (13.64–17.90)	0.14
Animal-based MUFA intake (g/day)	9.66 (7.66–11.78)	9.33 (7.04–11.00)	<0.05
Plant-based MUFA intake (g/day)	6.00 (4.77–7.36)	5.86 (4.53–7.06)	0.05
Total PUFA intake (g/day)	6.85 (5.78–8.18)	6.81 (5.77–7.97)	0.45
Total omega-3 PUFA^c^ intake (g/day)	0.80 (0.67–0.99)	0.81 (0.66–0.97)	0.88
Total omega-6 PUFA^d^ intake (g/day)	6.12 (5.03–7.26)	6.01 (5.07–6.97)	0.41
Marine omega-3 PUFA^e^ intake (g/day)	0.06 (0.03–0.11)	0.06 (0.04–0.10)	0.61
Omega-6/omega-3 ratio intake	7.47 (6.73–8.45)	7.32 (6.68–8.15)	0.09

FA, fatty acid; IQR, interquartile range; MET, metabolic equivalent task; MUFA, monounsaturated fatty acid; PUFA, polyunsaturated fatty acid; SFA, saturated fatty acid.

* *P* values were determined with Kruskal–Wallis test for continuous variables and Chi-square test for categorical variables. All statistical tests are two sided.

** Mean score of cases and controls: 593.3 versus 548.0.

† Adjusted for energy by the residual method except for total energy intake.

a Short-to-medium-chain SFA included saturated butyric (C4), caproic (C6), caprylic (C8), capric (C10), undecanoic (C11), lauric (C12), and tridecanoic (C13) acids.

b Long-chain SFA included saturated myristic (C14), pentadecanoic (C15), palmitic (C16), heptadecanoic (C17), stearic (C18), nonadecanoic (C19), arachidic (C20), behenic (C22), and lignoceric (C24) acids.

c Total omega-3 PUFA included alpha-linolenic acid, parinaric acid, docosatrienoic acid, eicosapentaenoic acid (EPA), docosapentaenoic acid (DPA), and docosahexaenoic acid (DHA).

d Total omega-6 PUFA included linoleic acid, eicosadienoic acid, arachidonic acid, and docosatetraenoic acid.

e Marine omega-3 PUFA included eicosapentaenoic acid (EPA), docosapentaenoic acid (DPA), and docosahexaenoic acid (DHA).

### Fat, FA, and asthenozoospermia

The correlations of dietary fat and FA consumptions with the odds of asthenozoospermia are displayed in Table 2. Comparing the highest with the lowest tertile of consumptions, plant-based fat was related to reduced odds of asthenozoospermia (OR = 0.68, 95% CI = 0.50–0.92, *P* trend <0.05), whereas animal-based MUFA was correlated to increased asthenozoospermia odds (OR = 1.49, 95% CI = 1.04–2.14, *P* trend <0.05), with significant dose–response relations (plant-based fat: OR = 0.85, 95% CI = 0.75–0.97; animal-based MUFA: OR = 1.24, 95% CI = 1.05–1.45; per SD increment). Moreover, per SD increment in omega-6 PUFA (OR = 0.86, 95% CI = 0.74-0.99) was correlated to 14% lower odds of asthenozoospermia. Null significant correlations were noticed between other fat and FA intakes and the odds of asthenozoospermia (Table 2). No significant curvilinear relation was observed between fat and FA consumptions and the odds of asthenozoospermia (Supplementary Figs S1, S2, S3, and S4).

**Table 2.** Adjusted odds ratios and 95% CIs for asthenozoospermia by tertile of dietary fat and fatty acid intake.

Variables	Cases (N = 549)	Controls (N = 581)	Multivariable-adjusted models		
			Model 1	Model 2	Model 3
**Total fat (g/day)** ^†^					
T1 (<46.60)	185	193	1.00 (Ref)	1.00 (Ref)	1.00 (Ref)
T2 (46.60 to <53.56)	178	193	1.02 (0.76–1.36)	1.08 (0.80–1.45)	0.97 (0.70–1.33)
T3 (≥53.56)	186	195	1.01 (0.76–1.35)	1.08 (0.80–1.45)	0.84 (0.56–1.27)
Continuous (per SD increment)			1.00 (0.89–1.13)	1.03 (0.91–1.16)	0.89 (0.73–1.08)
*P* for trend*			0.95	0.62	0.40
**Animal-based fat (g/day)** ^†^					
T1 (<24.23)	203	193	1.00 (Ref)	1.00 (Ref)	1.00 (Ref)
T2 (24.23 to <30.15)	177	193	1.12 (0.84–1.50)	1.10 (0.82–1.47)	1.07 (0.78–1.45)
T3 (≥30.15)	169	195	1.25 (0.94–1.67)	1.27 (0.95–1.71)	1.19 (0.82–1.73)
Continuous (per SD increment)			1.13 (1.00–1.27)	1.14 (1.01–1.28)	1.14 (0.96–1.36)
*P* for trend*			0.13	0.10	0.37
**Plant-based fat (g/day)** ^†^					
T1 (<20.12)	156	193	1.00 (Ref)	1.00 (Ref)	1.00 (Ref)
T2 (20.12 to <24.85)	169	193	0.83 (0.52–1.32)	0.91 (0.67–1.24)	0.90 (0.66–1.23)
T3 (≥24.85)	224	195	0.67 (0.50–0.90)	0.72 (0.53–0.97)	0.68 (0.50–0.92)
Continuous (per SD increment)			0.85 (0.76–0.96)	0.87 (0.77–0.99)	0.85 (0.75–0.97)
*P* for trend*			<0.05	<0.05	<0.05
**Total FA (g/day)** ^†^					
T1 (<35.15)	196	193	1.00 (Ref)	1.00 (Ref)	1.00 (Ref)
T2 (35.15 to <41.49)	170	193	1.12 (0.83–1.49)	1.13 (0.84–1.51)	1.05 (0.77–1.43)
T3 (≥41.49)	183	195	1.08 (0.81–1.44)	1.13 (0.84–1.51)	0.96 (0.66–1.39)
Continuous (per SD increment)			1.07 (0.95–1.21)	1.10 (0.97–1.24)	1.05 (0.89–1.24)
*P* for trend*			0.61	0.42	0.83
**Total SFA (g/day)** ^†^					
T1 (<13.87)	211	193	1.00 (Ref)	1.00 (Ref)	1.00 (Ref)
T2 (13.87 to <16.60)	172	193	1.17 (0.87–1.56)	1.20 (0.90–1.61)	1.16 (0.85–1.57)
T3 (≥16.60)	166	195	1.25 (0.94–1.66)	1.33 (0.99–1.78)	1.24 (0.89–1.73)
Continuous (per SD increment)			1.10 (0.98–1.24)	1.13 (1.00–1.28)	1.10 (0.96–1.27)
*P* for trend*			0.13	0.06	0.21
**Short-to-medium-chain SFA** ^a^ **(g/day)**^†^					
T1 (<0.56)	172	193	1.00 (Ref)	1.00 (Ref)	1.00 (Ref)
T2 (0.56 to <0.84)	173	193	0.92 (0.68–1.24)	0.97 (0.72–1.32)	0.97 (0.72–1.32)
T3 (≥0.84)	204	195	0.78 (0.58–1.04)	0.84 (0.62–1.13)	0.83 (0.61–1.11)
Continuous (per SD increment)			0.93 (0.83–1.05)	0.96 (0.85–1.09)	0.95 (0.84–1.08)
*P* for trend*			0.09	0.23	0.18
**Long-chain SFA** ^b^ **(g/day)**^†^					
T1 (<13.80)	217	193	1.00 (Ref)	1.00 (Ref)	1.00 (Ref)
T2 (13.80 to <16.38)	167	193	1.23 (0.92–1.65)	1.25 (0.94–1.68)	1.22 (0.90–1.66)
T3 (≥16.38)	165	195	1.32 (0.99–1.76)	1.38 (1.03–1.85)	1.31 (0.93–1.85)
Continuous (per SD increment)			1.13 (1.00–1.27)	1.16 (1.02–1.31)	1.14 (0.98–1.32)
*P* for trend*			0.06	<0.05	0.12
**Total MUFA (g/day)** ^†^					
T1 (<14.26)	211	193	1.00 (Ref)	1.00 (Ref)	1.00 (Ref)
T2 (14.26 to <16.96)	170	193	1.22 (0.91–1.62)	1.24 (0.93–1.66)	1.20 (0.88–1.63)
T3 (≥16.96)	168	195	1.28 (0.96–1.71)	1.31 (0.98–1.75)	1.21 (0.85–1.73)
Continuous (per SD increment)			1.09 (0.97–1.23)	1.11 (0.98–1.25)	1.07 (0.91–1.25)
*P* for trend*			0.09	0.07	0.28
**Animal-based MUFA (g/day)** ^†^					
T1 (<8.12)	215	193	1.00 (Ref)	1.00 (Ref)	1.00 (Ref)
T2 (8.12 to <10.68)	187	193	1.11 (0.84–1.47)	1.09 (0.82–1.46)	1.09 (0.81–1.47)
T3 (≥10.68)	147	195	1.50 (1.12–2.01)	1.49 (1.11–2.00)	1.49 (1.04–2.14)
Continuous (per SD increment)			1.21 (1.07–1.36)	1.20 (1.06–1.36)	1.24 (1.05–1.45)
*P* for trend*			<0.05	<0.05	<0.05
**Plant-based MUFA (g/day)** ^†^					
T1 (<5.15)	165	193	1.00 (Ref)	1.00 (Ref)	1.00 (Ref)
T2 (5.15 to <6.70)	179	193	0.87 (0.64–1.17)	0.91 (0.67–1.24)	0.91 (0.66–1.23)
T3 (≥6.70)	205	195	0.79 (0.59–1.06)	0.84 (0.63–1.13)	0.81 (0.60–1.10)
Continuous (per SD increment)			0.89 (0.79–1.00)	0.91 (0.80–1.02)	0.89 (0.79–1.01)
*P* for trend*			0.12	0.26	0.17
**Total PUFA (g/day)** ^†^					
T1 (<6.16)	193	193	1.00 (Ref)	1.00 (Ref)	1.00 (Ref)
T2 (6.16 to <7.62)	152	193	1.24 (0.92–1.68)	1.28 (0.94–1.74)	1.21 (0.88–1.65)
T3 (≥7.62)	204	195	0.98 (0.74–1.30)	1.02 (0.76–1.36)	0.87 (0.62–1.23)
Continuous (per SD increment)			0.95 (0.84–1.07)	0.96 (0.85–1.09)	0.86 (0.73–1.01)
*P* for trend*			0.78	0.96	0.37
**Total omega-3 PUFA** ^c^ **(g/day)**^†^					
T1 (<0.72)	194	193	1.00 (Ref)	1.00 (Ref)	1.00 (Ref)
T2 (0.72 to <0.90)	151	193	1.27 (0.94–1.71)	1.26 (0.93–1.71)	1.17 (0.85–1.62)
T3 (≥0.90)	204	195	1.00 (0.75–1.32)	1.03 (0.77–1.37)	0.87 (0.61–1.25)
Continuous (per SD increment)			1.02 (0.90–1.14)	1.02 (0.91–1.15)	0.94 (0.78–1.12)
*P* for trend*			0.86	0.97	0.38
**Total omega-6 PUFA** ^d^ **(g/day)**^†^					
T1 (<5.44)	190	193	1.00 (Ref)	1.00 (Ref)	1.00 (Ref)
T2 (5.44 to <6.71)	146	193	1.26 (0.93–1.71)	1.33 (0.97–1.82)	1.25 (0.91–1.73)
T3 (≥6.71)	213	195	0.92 (0.69–1.22)	0.96 (0.72–1.28)	0.81 (0.58–1.13)
Continuous (per SD increment)			0.94 (0.83–1.06)	0.95 (0.84–1.07)	0.86 (0.74–0.99)
*P* for trend*			0.42	0.57	0.15
**Marine omega-3 PUFA** ^e^ **(g/day)**^†^					
T1 (<0.04)	195	193	1.00 (Ref)	1.00 (Ref)	1.00 (Ref)
T2 (0.04 to <0.09)	176	193	1.11 (0.83–1.49)	1.08 (0.81–1.44)	1.05 (0.78–1.41)
T3 (≥0.09)	178	195	1.17 (0.88–1.57)	1.13 (0.84–1.51)	1.08 (0.80–1.47)
Continuous (per SD increment)			1.09 (0.97–1.23)	1.08 (0.96–1.22)	1.06 (0.93–1.21)
*P* for trend*			0.30	0.43	0.62
**Omega-6/omega-3 ratio** ^†^					
T1 (<6.91)	173	193	1.00 (Ref)	1.00 (Ref)	1.00 (Ref)
T2 (6.91 to <7.92)	158	193	1.08 (0.80–1.45)	1.09 (0.81–1.48)	1.10 (0.81–1.49)
T3 (≥7.92)	218	195	0.77 (0.58–1.02)	0.78 (0.59–1.04)	0.79 (0.59–1.08)
Continuous (per SD increment)			0.92 (0.82–1.04)	0.93 (0.82–1.05)	0.94 (0.83–1.07)
*P* for trend*			<0.05	0.06	0.09

FA, fatty acid; MUFA, monounsaturated fatty acid; PUFA, polyunsaturated fatty acid; Ref, reference; SFA, saturated fatty acid; T, tertile.

The SD for the listed fat and FAs are 8.75, 8.12, 6.83, 8.17, 3.66, 0.40, 3.59, 3.62, 3.31, 2.48, 2.22, 0.29, 1.99, 0.07, and 1.69 g/day, respectively.

Model 1: adjusted for age (continuous, years) and total energy intake (continuous, kcal/day).

Model 2: same as Model 1 and further adjusted for BMI (continuous, kg/m^2^), alcohol drinking (yes or no), cigarette smoking (yes or no), dietary change (yes or no), household income (<50, 50 to <100, or ≥100, thousand yuan), education (junior secondary or below, senior high school/technical secondary school, and junior college/university or above), physical activity (continuous, metabolic equivalent/hours/week), and abstinence time (continuous, days).

Model 3: same as Model 2 and further adjusted for total protein (continuous, g/day) and total carbohydrate (continuous, g/day) intake.

† Adjusted for energy by the residual method.

* *P* value for linear trend calculated from category median values.

a Short-to-medium-chain SFA included saturated butyric (C4), caproic (C6), caprylic (C8), capric (C10), undecanoic (C11), lauric (C12), and tridecanoic (C13) acids.

b Long-chain SFA included saturated myristic (C14), pentadecanoic (C15), palmitic (C16), heptadecanoic (C17), stearic (C18), nonadecanoic (C19), arachidic (C20), behenic (C22), and lignoceric (C24) acids.

c Total omega-3 PUFA included alpha-linolenic acid, parinaric acid, docosatrienoic acid, eicosapentaenoic acid (EPA), docosapentaenoic acid (DPA), and docosahexaenoic acid (DHA).

d Total omega-6 PUFA included linoleic acid, eicosadienoic acid, arachidonic acid, and docosatetraenoic acid.

e Marine omega-3 PUFA included eicosapentaenoic acid (EPA), docosapentaenoic acid (DPA), and docosahexaenoic acid (DHA).

Among the common FAs, we found that higher consumptions of stearic acid (OR = 1.74, 95% CI = 1.21–2.53, *P* trend <0.05), palmitoleic acid (OR = 1.50, 95% CI = 1.05–2.16, *P* trend <0.05), and arachidonic acid (OR = 1.45, 95% CI = 1.03–2.05, *P* trend <0.05) were positively correlated to asthenozoospermia odds, and significant dose–response relations were also observed (stearic acid: OR = 1.29, 95% CI = 1.09–1.52; palmitoleic acid: OR = 1.29, 95% CI = 1.09–1.54; arachidonic acid: OR = 1.21, 95% CI = 1.03–1.42; per SD increment) (Table 3). In addition, linoleic acid consumption was related to dose–response decreased asthenozoospermia odds (OR = 0.86, 95% CI = 0.74-0.99, per SD increment) (Table 3).

**Table 3.** Adjusted odds ratios and 95% CIs for asthenozoospermia by tertile of dietary common fatty acid intake.*

Variables		Cases (N = 549)	Controls (N = 581)	OR (95% CI)
**Capric acid (g/day)** ^†^	T1 (<0.14)	174	193	1.00 (Ref)
	T2 (0.14 to <0.21)	165	193	0.98 (0.73–1.33)
	T3 (≥0.21)	210	195	0.78 (0.58–1.06)
	Continuous (per SD increment)			0.91 (0.80–1.03)
	*P* for trend**			0.09
**Lauric acid (g/day)** ^†^	T1 (<0.35)	173	193	1.00 (Ref)
	T2 (0.35 to <0.51)	177	193	0.95 (0.70–1.28)
	T3 (≥0.51)	199	195	0.85 (0.63–1.14)
	Continuous (per SD increment)			0.97 (0.86–1.10)
	*P* for trend**			0.26
**Myristic acid (g/day)** ^†^	T1 (<0.85)	203	193	1.00 (Ref)
	T2 (0.85 to <1.16)	169	193	1.15 (0.86–1.56)
	T3 (≥1.16)	177	195	1.17 (0.86–1.60)
	Continuous (per SD increment)			1.05 (0.93–1.20)
	*P* for trend**			0.34
**Palmitic acid (g/day)** ^†^	T1 (<9.41)	205	193	1.00 (Ref)
	T2 (9.41 to <11.14)	179	193	1.04 (0.76–1.41)
	T3 (≥11.14)	165	195	1.16 (0.84–1.62)
	Continuous (per SD increment)			1.07 (0.94–1.23)
	*P* for trend**			0.37
**Stearic acid (g/day)** ^†^	T1 (<3.05)	217	193	1.00 (Ref)
	T2 (3.05 to <3.77)	195	193	1.14 (0.85–1.55)
	T3 (≥3.77)	137	195	1.74 (1.21–2.53)
	Continuous (per SD increment)			1.29 (1.09–1.52)
	*P* for trend**			<0.05
**Arachidic acid (g/day)** ^†^	T1 (<0.07)	200	193	1.00 (Ref)
	T2 (0.07 to <0.09)	193	193	0.98 (0.72–1.33)
	T3 (≥0.09)	156	195	1.26 (0.86–1.84)
	Continuous (per SD increment)			1.16 (0.98–1.39)
	*P* for trend**			0.21
**Palmitoleic acid (g/day)** ^†^	T1 (<0.71)	225	193	1.00 (Ref)
	T2 (0.71 to <0.94)	167	193	1.35 (0.99–1.83)
	T3 (≥0.94)	157	195	1.50 (1.05–2.16)
	Continuous (per SD increment)			1.29 (1.09–1.54)
	*P* for trend**			<0.05
**Oleic acid (g/day)** ^†^	T1 (<12.41)	210	193	1.00 (Ref)
	T2 (12.41 to <14.83)	165	193	1.19 (0.87–1.62)
	T3 (≥14.83)	174	195	1.14 (0.80–1.61)
	Continuous (per SD increment)			1.04 (0.89–1.22)
	*P* for trend**			0.47
**Erucic acid (g/day)** ^†^	T1 (<0.13)	197	193	1.00 (Ref)
	T2 (0.13 to <0.29)	167	193	1.11 (0.80–1.54)
	T3 (≥0.29)	185	195	1.09 (0.81–1.46)
	Continuous (per SD increment)			0.95 (0.84–1.07)
	*P* for trend**			0.67
**Linoleic acid (g/day)** ^†^	T1 (<5.39)	188	193	1.00 (Ref)
	T2 (5.39 to <6.66)	147	193	1.22 (0.89–1.69)
	T3 (≥6.66)	214	195	0.80 (0.57–1.11)
	Continuous (per SD increment)			0.86 (0.74–0.99)
	*P* for trend**			0.12
**α-Linolenic acid (g/day)** ^†^	T1 (<0.63)	187	193	1.00 (Ref)
	T2 (0.63 to <0.81)	163	193	1.06 (0.77–1.46)
	T3 (≥0.81)	199	195	0.86 (0.60–1.22)
	Continuous (per SD increment)			0.91 (0.77–1.08)
	*P* for trend**			0.33
**Arachidonic acid (g/day)** ^†^	T1 (<0.03)	229	193	1.00 (Ref)
	T2 (0.03 to <0.04)	162	193	1.38 (1.01–1.87)
	T3 (≥0.04)	158	195	1.45 (1.03–2.05)
	Continuous (per SD increment)			1.21 (1.03–1.42)
	*P* for trend**			<0.05
**Eicosapentaenoic acid (g/day)** ^†^	T1 (<0.03)	196	193	1.00 (Ref)
	T2 (0.03 to <0.05)	178	193	1.06 (0.78–1.42)
	T3 (≥0.05)	175	195	1.09 (0.80–1.49)
	Continuous (per SD increment)			1.08 (0.95–1.23)
	*P* for trend**			0.59
**Docosahexaenoic acid (g/day)** ^†^	T1 (<0.01)	194	193	1.00 (Ref)
	T2 (0.01 to <0.04)	174	193	1.07 (0.80–1.44)
	T3 (≥0.04)	181	195	1.07 (0.79–1.45)
	Continuous (per SD increment)			1.04 (0.92–1.18)
	*P* for trend**			0.71

MET, metabolic equivalent; OR, odds ratio; Ref, reference; T, tertile.

The SD for the listed fatty acids are 0.10, 0.26, 0.41, 2.66, 0.99, 0.03, 0.28, 3.27, 0.44, 1.99, 0.27, 0.01, 0.03, and 0.03 g/day, respectively.

* Odds ratios and 95% CIs were calculated with the use of unconditional logistic regression model with adjustment for age (continuous, years), BMI (continuous, kg/m^2^), alcohol drinking (yes or no), cigarette smoking (yes or no), dietary change (yes or no), household income (<50, 50 to <100, or ≥100, thousand yuan), education (junior secondary or below, senior high school/technical secondary school, and junior college/university or above), physical activity (continuous, MET/hours/week), abstinence time (continuous, days), and total energy (continuous, kcal/day), total protein (continuous, g/day), and total carbohydrate (continuous, g/day) intake.

† Adjusted for energy by the residual method.

** Test for trend based on variables containing the median value for each tertile.

### Subgroup, interaction, and sensitivity analyses

The associations between fat and FA intakes and the odds of asthenozoospermia were consistent with the primary results across different subgroups (Supplementary Figs S5, S6, S7, S8 and S9). Notably, we found higher consumptions of total SFA, long-chain SFA, and total MUFA were correlated to increased asthenozoospermia odds in the subgroup of non-drinkers (Supplementary Figs S6 and S7). A similar pattern was noticed between long-chain SFA intake and the odds of asthenozoospermia in the subgroup of age <32 years (Supplementary Fig. S6). Conversely, plant-based MUFA consumption was negatively correlated to the odds of asthenozoospermia in the subgroup of non-smokers (Supplementary Fig. S7). Furthermore, we observed statistically significant multiplicative interactions of animal-based fat and animal-based MUFA intake with cigarette smoking and marine omega-3 PUFA intake with alcohol drinking on the odds of asthenozoospermia (Supplementary Figs S5, S7, and S9). In addition, significant additive interactions were found between animal-based MUFA and total FA intake with cigarette smoking and animal-based MUFA intake with alcohol drinking on the odds of asthenozoospermia (Supplementary Table S1). However, no significant additive interactions were noticed between fat and FAs intake and other stratified variables (Supplementary Table S2).

In the sensitivity analyses, results did not change substantially when adjusted for energy intake with the nutrient-density method or when excluding the participants with dietary change compared with the original analyses (Supplementary Tables S3 and S4).

## Discussion

Our findings first demonstrated that the consumption of plant-based fat was correlated to decreased asthenozoospermia odds, whereas animal-based MUFA intake was positively related to increased odds of asthenozoospermia. Among the common FAs, stearic acid, palmitoleic acid, and arachidonic acid intake were associated with increased odds of asthenozoospermia. Of note, interaction analyses on both multiplicative and additive scales suggested that cigarette smoking might modify the relation of animal-based MUFA with the odds of asthenozoospermia in a negative manner.

Evidence from prior research on the relations of dietary FA consumptions with the odds of asthenozoospermia has been limited. One previous study indicated that a nutrient pattern that was abundant in PUFA, fiber, vitamins, and minerals was correlated to reduced risk of asthenozoospermia (Eslamian *et al.*, 2017). Eslamian *et al.* conducted a case–control study with 107 asthenozoospermia cases and 235 age-matched controls, where they found that SFA, TFA, stearic acid, and palmitic acid intakes were positively related to the odds of asthenozoospermia, whereas higher consumptions of DHA and omega-3 PUFA were significantly correlated to decreased asthenozoospermia odds (Eslamian *et al.*, 2015). These inconsistencies might be attributable to different consumptions of FAs, exposure assessment, sample sizes, dietary habits, and potential confounders adjustment. For instance, the median consumptions of SFA, omega-3 PUFA, and DHA in the study of Eslamian *et al.* (2015) were obviously higher than that in our study. Moreover, the differences in dietary habits and different numbers of FFQ food items might affect the assessment of dietary FA intake. Additionally, a larger sample size in the present study could provide relatively higher statistical efficiency for these estimates. Furthermore, age, alcohol drinking, household income, and physical activity might be associated with sperm quality; however, these potential confounders were not adjusted in the study of Eslamian *et al.* (2015).

Recent evidence indicated that saturated fat was related to a lower total sperm count and sperm concentration, and higher monounsaturated fat consumption was related to a reduced number of sperm with normal morphology (Jensen *et al.*, 2013). Furthermore, Attaman *et al.* (2012) found that higher consumption of total fat was correlated to lower sperm concentration and total sperm count. These above studies revealed that total fat, saturated fat, and monounsaturated fat intakes were correlated to lower sperm quality and more likely to be related to the odds of asthenozoospermia. Nevertheless, Povey *et al.* (2020) found that some high saturated fat foods, including full-fat milk, red meat, and butter, were related to better sperm quality parameters, which suggested that the association of dietary fat and sperm quality may be related to different food sources. Previous studies also indicated that different sources of fat and FA exerted different effects on health outcomes (Rice *et al.*, 2020; Abbate *et al.*, 2021; Van Blarigan *et al.*, 2023), but no studies have probed into the effect of different sources of fat and FA on asthenozoospermia or sperm quality so far. Hence, we explored the relation between dietary fat and asthenozoospermia odds and further considered the effect of different food sources of fat. Nevertheless, given the limited evidence on dietary fat and FA consumptions and asthenozoospermia odds, further research is needed to confirm our findings.

More importantly, we identified significant interactions on both additive and multiplicative scales between animal-based MUFA intake and cigarette smoking on the odds of asthenozoospermia, suggesting that smoking might synergistically interact with animal-based MUFA intake to further increase the odds of asthenozoospermia. Although no previous studies have investigated this topic, this interaction is potentially supported by several molecular mechanisms. The results of sperm metabolomic analysis have revealed that smoking could reduce the long-chain FA uptake of sperm mitochondria and impair the energy supply of sperm, leading to poor sperm quality (Engel *et al.*, 2021). Moreover, unsaturated FAs are a significant part of the sperm membrane, which may exert a crucial role in maintaining the normal function of sperm (Agarwal *et al.*, 2006). Cigarette smoking may increase lipid peroxidation and malondialdehyde in sperm via the production of nitric oxide and other oxidative agents, which could result in the oxidative damage of unsaturated FAs in sperm membranes and lead to poor sperm quality (Ghaffari and Rostami, 2012). Restricted to the limited evidence, the possibility of incidental discovery could not be fully eliminated; thus, additional studies on the interaction between unsaturated FAs and smoking on the odds of asthenozoospermia are required.

Although the etiology of dietary fat and FAs in relation to the odds of asthenozoospermia is not yet clear, there have been several possible explanations for our findings. On the one hand, plant-based fat is abundant in vitamin E, micronutrients, and phytochemicals, which have been proven to be related to better sperm quality (Mínguez-Alarcón *et al.*, 2012; De Cosmi *et al.*, 2021; Talebi *et al.*, 2022). On the other hand, legume, vegetable, and whole grain (specific foods: bean products, soybean milk, soybean, tomato, corn, and leek) are the main sources of plant-based fat in the population of this study owing to the relatively high intake of these foods. These foods usually contain ample fiber, which can decrease the plasma estrogen level by binding directly to unconjugated estrogens (Hu, 2002) and lowering the deconjugating bacterial count (Eslamian *et al.*, 2016) in the gastrointestinal tract, leading to less reabsorption of estrogens and a lower asthenozoospermia risk. Furthermore, animal-based fat and FA are mainly derived from high-fat dairy products, processed meat, and meat, and these foods usually contain higher lipophilic-containing substances such as xenoestrogens (Carreau *et al.*, 2012). A previous study has shown that xenobiotics, particularly xenoestrogens, could bind to the sperm membrane, with their concentration having an inverse relation with sperm motility (Rozati *et al.*, 2002). Future research should further explore the impact of diverse types and sources of fat and FA on the odds of asthenozoospermia to illustrate the detailed biological mechanisms.

Several strengths of our research are worth mentioning. First of all, a validated FFQ was applied to estimate the fat and FA consumptions of the study population. Another distinctive feature of this analysis compared with previous studies is that we considered the different sources of fat and FA consumptions on the odds of asthenozoospermia and analyze the interactions between fat and FA intakes and several important variables on both additive and multiplicative scales. Additionally, the relatively large sample sizes and high participation rates of both case and control groups provided more reliable results. Furthermore, we rigorously controlled for important confounding factors and conducted multiple subgroup and sensitivity analyses, which further strengthened the robustness of our primary findings.

However, several limitations also must be addressed. First, recall bias is unavoidable in any case–control studies. However, a highly reproducible verified FFQ and experienced personnel could alleviate this concern and provide more reliable dietary data. Second, the participants are not a random sample of the whole target population, which might result in selection bias. To minimize this source of possible bias and improve the comparability of cases and controls, we selected the controls from the same infertility clinic and controlled for crucial demographic characteristics variables. Third, we failed to obtain the consumption of TFA, and as a result, the correlation of TFA with asthenozoospermia odds was unclear. Nevertheless, the consumption of TFA in the Chinese population remained at a relatively low level compared with the WHO-recommended level and that in other countries (Jiang *et al.*, 2020). Fourth, although a diversity of confounding factors was considered, residual confounding factors could not be completely ruled out in any of the case–control studies. Fifth, owing to the multicollinearity of ultra-processed foods and meat with dietary fat and FA, we failed to adjust for them in the final model, therefore potential residual confounding of these food items on the relations of dietary fat and FA intakes with the odds of asthenozoospermia might not have been eliminated thoroughly. Finally, as the current study only included the Chinese population, our findings should be interpreted with caution when generalizing to other populations.

## Conclusion

This study has added novel evidence to the existing knowledge about dietary fat and FA intakes and asthenozoospermia odds. In summary, our results suggested that plant-based fat consumption was inversely correlated to asthenozoospermia odds, whereas a positive relation was observed between animal-based MUFA consumption and the odds of asthenozoospermia. These findings highlight the possibility that increasing the consumption of plant-derived fat and FA and decreasing animal-derived fat and FA intakes might be beneficial in the prevention of asthenozoospermia. These reported relations need to be supported by further large-scale prospective cohort studies and clinical trials.

## Supplementary Material

hoad030_Supplementary_Figures

hoad030_Supplementary_Tables

## Data Availability

The data that support the findings of our study are available from the corresponding author upon reasonable request.

## References

[hoad030-B1] Abbate M , Mascaró CM, Montemayor S, Barbería-Latasa M, Casares M, Gómez C, Ugarriza L, Tejada S, Abete I, Zulet MÁ et al Animal Fat Intake Is Associated with Albuminuria in Patients with Non-Alcoholic Fatty Liver Disease and Metabolic Syndrome. Nutrients 2021;13:1548. 34064372 10.3390/nu13051548 PMC8147815

[hoad030-B2] Adams JA , Galloway TS, Mondal D, Esteves SC, Mathews F. Effect of mobile telephones on sperm quality: a systematic review and meta-analysis. Environ Int 2014;70:106–112. 24927498 10.1016/j.envint.2014.04.015

[hoad030-B3] Agarwal A , Gupta S, Sikka S. The role of free radicals and antioxidants in reproduction. Curr Opin Obstet Gynecol 2006;18:325–332. 16735834 10.1097/01.gco.0000193003.58158.4e

[hoad030-B4] Ainsworth BE , Haskell WL, Herrmann SD, Meckes N, Bassett DR Jr, Tudor-Locke C, Greer JL, Vezina J, Whitt-Glover MC, Leon AS. 2011 compendium of physical activities: a second update of codes and MET values. Med Sci Sports Exerc 2011;43:1575–1581. 21681120 10.1249/MSS.0b013e31821ece12

[hoad030-B5] Attaman JA , Toth TL, Furtado J, Campos H, Hauser R, Chavarro JE. Dietary fat and semen quality among men attending a fertility clinic. Hum Reprod 2012;27:1466–1474. 22416013 10.1093/humrep/des065 PMC3329193

[hoad030-B6] Benoff S , Auborn K, Marmar JL, Hurley IR. Link between low-dose environmentally relevant cadmium exposures and asthenozoospermia in a rat model. Fertil Steril 2008;89:e73-79–e79. 18308070 10.1016/j.fertnstert.2007.12.035 PMC2567823

[hoad030-B7] Boivin J , Bunting L, Collins JA, Nygren KG. International estimates of infertility prevalence and treatment-seeking: potential need and demand for infertility medical care. Hum Reprod 2007;22:1506–1512. 17376819 10.1093/humrep/dem046

[hoad030-B8] Bonde JP , Ernst E, Jensen TK, Hjollund NH, Kolstad H, Henriksen TB, Scheike T, Giwercman A, Olsen J, Skakkebaek NE. Relation between semen quality and fertility: a population-based study of 430 first-pregnancy planners. Lancet (London, England) 1998;352:1172–1177. 9777833 10.1016/S0140-6736(97)10514-1

[hoad030-B9] Cai T , Wagenlehner FM, Mondaini N, D'Elia C, Meacci F, Migno S, Malossini G, Mazzoli S, Bartoletti R. Effect of human papillomavirus and Chlamydia trachomatis co-infection on sperm quality in young heterosexual men with chronic prostatitis-related symptoms. BJU Int 2014;113:281–287. 23906072 10.1111/bju.12244

[hoad030-B10] Calder PC. Functional roles of fatty acids and their effects on human health. JPEN J Parenter Enteral Nutr 2015;39:18s–32s. 26177664 10.1177/0148607115595980

[hoad030-B11] Carreau S , Bouraima-Lelong H, Delalande C. Role of estrogens in spermatogenesis. Front Biosci (Elite Ed.) 2012;4:1–11. 22201851 10.2741/e356

[hoad030-B12] Cui Q , Wang HH, Wu QJ, Wang XB, Guo RH, Leng X, Tan XL, Du Q, Pan BC. Diet quality scores and asthenoteratozoospermia risk: finding from a hospital-based case-control study in china. Front Nutr 2022a;9:859143. 35479758 10.3389/fnut.2022.859143 PMC9036176

[hoad030-B13] Cui Q , Xia Y, Liu YS, Sun YF, Ye K, Li WJ, Wu QJ, Chang Q, Zhao YH. Validity and reproducibility of a FFQ for assessing dietary intake among residents of northeast China: northeast cohort study of China. Br J Nutr 2022b;129:1252–1265. 10.1017/S0007114522002318 35912695

[hoad030-B14] Curi SM , Ariagno JI, Chenlo PH, Mendeluk GR, Pugliese MN, Sardi Segovia LM, Repetto HE, Blanco AM. Asthenozoospermia: analysis of a large population. Arch Androl 2003;49:343–349. 12893510 10.1080/01485010390219656

[hoad030-B15] De Cosmi V , Parazzini F, Agostoni C, Noli S, Cipriani S, La Vecchia I, Ferrari S, Esposito G, Bravi F, Ricci E. Antioxidant vitamins and carotenoids intake and the association with poor semen quality: a cross-sectional analysis of men referring to an Italian Fertility Clinic. Front Nutr 2021;8:737077. 34671631 10.3389/fnut.2021.737077 PMC8520935

[hoad030-B16] Du H , Bennett D, Li L, Whitlock G, Guo Y, Collins R, Chen J, Bian Z, Hong LS, Feng S et al; China Kadoorie Biobank Collaborative Group. Physical activity and sedentary leisure time and their associations with BMI, waist circumference, and percentage body fat in 0.5 million adults: the China Kadoorie Biobank study. Am J Clin Nutr 2013;97:487–496. 23364014 10.3945/ajcn.112.046854 PMC4345799

[hoad030-B17] Efrat M , Stein A, Pinkas H, Unger R, Birk R. Dietary patterns are positively associated with semen quality. Fertil Steril 2018;109:809–816. 29778381 10.1016/j.fertnstert.2018.01.010

[hoad030-B18] Engel KM , Baumann S, Blaurock J, Rolle-Kampczyk U, Schiller J, von Bergen M, Grunewald S. Differences in the sperm metabolomes of smoking and nonsmoking men. Biol Reprod 2021;105:1484–1493. 34554205 10.1093/biolre/ioab179

[hoad030-B19] Eslamian G , Amirjannati N, Noori N, Sadeghi MR, Hekmatdoost A. Effects of coadministration of DHA and vitamin E on spermatogram, seminal oxidative stress, and sperm phospholipids in asthenozoospermic men: a randomized controlled trial. Am J Clin Nutr 2020;112:707–719. 32453396 10.1093/ajcn/nqaa124

[hoad030-B20] Eslamian G , Amirjannati N, Rashidkhani B, Sadeghi MR, Baghestani AR, Hekmatdoost A. Dietary fatty acid intakes and asthenozoospermia: a case-control study. Fertil Steril 2015;103:190–198. 25456794 10.1016/j.fertnstert.2014.10.010

[hoad030-B21] Eslamian G , Amirjannati N, Rashidkhani B, Sadeghi MR, Baghestani AR, Hekmatdoost A. Adherence to the Western Pattern Is Potentially an Unfavorable Indicator of Asthenozoospermia Risk: A Case-Control Study. J Am Coll Nutr 2016;35:50–58. 25764357 10.1080/07315724.2014.936983

[hoad030-B22] Eslamian G , Amirjannati N, Rashidkhani B, Sadeghi MR, Hekmatdoost A. Intake of food groups and idiopathic asthenozoospermia: a case-control study. Hum Reprod 2012;27:3328–3336. 22940769 10.1093/humrep/des311

[hoad030-B23] Eslamian G , Amirjannati N, Rashidkhani B, Sadeghi M-R, Hekmatdoost A. Nutrient patterns and asthenozoospermia: a case-control study. Andrologia 2017;49:e12624. 10.1111/and.12624 27246740

[hoad030-B24] Ghaffari MA , Rostami M. Lipid peroxidation and nitric oxide levels in male smokers' spermatozoa and their relation with sperm motility. J Reprod Infertil 2012;13:81–87. 23926529 PMC3719338

[hoad030-B25] Govindarajulu US , Malloy EJ, Ganguli B, Spiegelman D, Eisen EA. The comparison of alternative smoothing methods for fitting non-linear exposure-response relationships with Cox models in a simulation study. Int J Biostat 2009;5:Article 2. 20231865 10.2202/1557-4679.1104 PMC2827890

[hoad030-B26] Hu FB. Dietary pattern analysis: a new direction in nutritional epidemiology. Curr Opin Lipidol 2002;13:3–9. 11790957 10.1097/00041433-200202000-00002

[hoad030-B27] Hu Y , Ding M, Yuan C, Wu K, Smith-Warner SA, Hu FB, Chan AT, Meyerhardt JA, Ogino S, Fuchs CS et al Association between coffee intake after diagnosis of colorectal cancer and reduced mortality. Gastroenterology 2018;154:916–926.e919. 29158191 10.1053/j.gastro.2017.11.010 PMC5847429

[hoad030-B28] Huang DH , Zhang YX, Wang XB, Guo RH, Leng X, Du Q, Wu QJ, Pan BC, Zhao YH. Dietary total antioxidant capacity and the risk of developing asthenozoospermia: a hospital-based case-control study in China. Hum Reprod 2023;38:537–548. 36728412 10.1093/humrep/dead010

[hoad030-B29] Jensen TK , Heitmann BL, Blomberg Jensen M, Halldorsson TI, Andersson AM, Skakkebæk NE, Joensen UN, Lauritsen MP, Christiansen P, Dalgård C et al High dietary intake of saturated fat is associated with reduced semen quality among 701 young Danish men from the general population. Am J Clin Nutr 2013;97:411–418. 23269819 10.3945/ajcn.112.042432

[hoad030-B30] Jiang LY , Shen JJ, Zhao YX, Li JW, Liu SN, Liu YJ, Wang HJ, Su C, Zhuang X, Chen NH et al Trans fatty acid intake among Chinese population: a longitudinal study from 1991 to 2011. Lipids Health Dis 2020;19:80. 32340620 10.1186/s12944-020-01247-1 PMC7184713

[hoad030-B31] Jóźków P , Mędraś M, Lwow F, Zagrodna A, Słowińska-Lisowska M. Associations between physical activity and semen quality in young healthy men. Fertil Steril 2017;107:373–378.e372. 27919439 10.1016/j.fertnstert.2016.11.004

[hoad030-B32] Kurtz MP , Zurakowski D, Rosoklija I, Bauer SB, Borer JG, Johnson KL, Migliozzi M, Diamond DA. Semen parameters in adolescents with varicocele: association with testis volume differential and total testis volume. J Urol 2015;193:1843–1847. 25813564 10.1016/j.juro.2014.10.111

[hoad030-B33] Levine H , Jørgensen N, Martino-Andrade A, Mendiola J, Weksler-Derri D, Jolles M, Pinotti R, Swan SH. Temporal trends in sperm count: a systematic review and meta-regression analysis of samples collected globally in the 20th and 21st centuries. Hum Reprod Update 2023;29:157–176. 36377604 10.1093/humupd/dmac035

[hoad030-B34] Li XY , Wang XB, Wu QJ, Guo RH, Leng X, Du Q, Pan BC, Zhao YH. Short total sleep duration and poor sleep quality might be associated with asthenozoospermia risk: a case-control study. Front Physiol 2022;13:959009. 36277203 10.3389/fphys.2022.959009 PMC9581216

[hoad030-B35] Liu FH , Wang XB, Wen ZY, Wang HY, Zhang M, Zhang S, Jiang YT, Zhang JY, Sun H, Pan BC et al Dietary inflammatory index and risk of asthenozoospermia: a hospital-based case-controlled study in China. Front Nutr 2021;8:706869. 34395499 10.3389/fnut.2021.706869 PMC8357981

[hoad030-B36] Liu Y-S , Zhang Y-X, Wang X-B, Wu Q-J, Liu F-H, Pan B-C, Zhao Y-H. Associations between Meat and Vegetable Intake, Cooking Methods, and Asthenozoospermia: A Hospital-Based Case–Control Study in China. Nutrients 2022;14:1956. 35565922 10.3390/nu14091956 PMC9104795

[hoad030-B37] Lv JL , Wu QJ, Wang XB, Du Q, Liu FH, Guo RH, Leng X, Pan BC, Zhao YH. Intake of ultra-processed foods and asthenozoospermia odds: a hospital-based case-control study. Front Nutr 2022;9:941745. 36337657 10.3389/fnut.2022.941745 PMC9630735

[hoad030-B38] Martini AC , Tissera A, Estofán D, Molina RI, Mangeaud A, de Cuneo MF, Ruiz RD. Overweight and seminal quality: a study of 794 patients. Fertil Steril 2010;94:1739–1743. 20056217 10.1016/j.fertnstert.2009.11.017

[hoad030-B39] Mínguez-Alarcón L , Mendiola J, López-Espín JJ, Sarabia-Cos L, Vivero-Salmerón G, Vioque J, Navarrete-Muñoz EM, Torres-Cantero AM. Dietary intake of antioxidant nutrients is associated with semen quality in young university students. Hum Reprod 2012;27:2807–2814. 22752607 10.1093/humrep/des247

[hoad030-B40] Patel AS , Leong JY, Ramasamy R. Prediction of male infertility by the World Health Organization laboratory manual for assessment of semen analysis: a systematic review. Arab J Urol 2018;16:96–102. 29713540 10.1016/j.aju.2017.10.005 PMC5922004

[hoad030-B41] Povey AC , Clyma JA, McNamee R, Moore HD, Baillie H, Pacey AA, Cade JE, Cherry NM; Participating Centres of Chaps-UK. Phytoestrogen intake and other dietary risk factors for low motile sperm count and poor sperm morphology. Andrology 2020;8:1805–1814. 32649041 10.1111/andr.12858

[hoad030-B42] Radzikowska U , Rinaldi AO, Çelebi Sözener Z, Karaguzel D, Wojcik M, Cypryk K, Akdis M, Akdis CA, Sokolowska M. The Influence of Dietary Fatty Acids on Immune Responses. Nutrients 2019;11:2990. 31817726 10.3390/nu11122990 PMC6950146

[hoad030-B43] Rice MS , Poole EM, Willett WC, Tworoger SS. Adult dietary fat intake and ovarian cancer risk. Int J Cancer 2020;146:2756–2772. 31443135 10.1002/ijc.32635 PMC7201382

[hoad030-B44] Rozati R , Reddy PP, Reddanna P, Mujtaba R. Role of environmental estrogens in the deterioration of male factor fertility. Fertil Steril 2002;78:1187–1194. 12477510 10.1016/s0015-0282(02)04389-3

[hoad030-B45] Skakkebæk NE , Lindahl-Jacobsen R, Levine H, Andersson AM, Jørgensen N, Main KM, Lidegaard Ø, Priskorn L, Holmboe SA, Bräuner EV et al Environmental factors in declining human fertility. Nat Rev Endocrinol 2022;18:139–157. 34912078 10.1038/s41574-021-00598-8

[hoad030-B46] Talebi S , Arab A, Sorraya N. The association between dietary antioxidants and semen parameters: a cross-sectional study among iranian infertile men. Biol Trace Elem Res 2022;200:3957–3964. 34741245 10.1007/s12011-021-03007-3

[hoad030-B47] Uauy R , Dangour AD. Fat and fatty acid requirements and recommendations for infants of 0-2 years and children of 2-18 years. Ann Nutr Metab 2009;55:76–96. 19752537 10.1159/000228997

[hoad030-B48] Uauy R , Hoffman DR, Peirano P, Birch DG, Birch EE. Essential fatty acids in visual and brain development. Lipids 2001;36:885–895. 11724460 10.1007/s11745-001-0798-1

[hoad030-B49] Van Blarigan EL , Ma C, Ou FS, Bainter TM, Venook AP, Ng K, Niedzwiecki D, Giovannucci E, Lenz HJ, Polite BN et al Dietary fat in relation to all-cause mortality and cancer progression and death among people with metastatic colorectal cancer: data from CALGB 80405 (Alliance)/SWOG 80405. Intl Journal of Cancer 2023;152:123–136. 10.1002/ijc.34230 PMC9691576 35904874

[hoad030-B50] Wang XB , Wu QJ, Guo RH, Leng X, Du Q, Zhao YH, Pan BC. Dairy product consumption and oligo-astheno-teratozoospermia risk: a hospital-based case-control study in China. Front Nutr 2021a;8:742375. 34993218 10.3389/fnut.2021.742375 PMC8724031

[hoad030-B51] Wang XB , Wu QJ, Liu FH, Zhang S, Wang HY, Guo RH, Leng X, Du Q, Zhao YH, Pan BC. The association between dairy product consumption and asthenozoospermia risk: a hospital-based case-control study. Front Nutr 2021b;8:714291. 34746202 10.3389/fnut.2021.714291 PMC8566545

[hoad030-B52] Whittaker J. Dietary trends and the decline in male reproductive health. Hormones (Athens, Greece) 2023;22:165–197. 36725796 10.1007/s42000-023-00431-z

[hoad030-B53] Willett W , Stampfer MJ. Total energy intake: implications for epidemiologic analyses. Am J Epidemiol 1986;124:17–27. 3521261 10.1093/oxfordjournals.aje.a114366

[hoad030-B54] World Health Organization. WHO Laboratory Manual for the Examination and Processing of Human Semen, 5th edn. Geneva, Switzerland: World Health Organization, 2010.

[hoad030-B55] World Health Organization. WHO Laboratory Manual for the Examination and Processing of Human Semen. Geneva: World Health Organization, 2021.

[hoad030-B56] Yang YX , Wang YG, He M, Pan XC, Wang Z. China Food Composition, Standard Edition. Beijing: Peking University Medical Press, 2018.

[hoad030-B57] Zhang HH , Xia Y, Chang Q, Gao SY, Zhao YH. Dietary patterns and associations between air pollution and gestational diabetes mellitus. Environment International 2021;147:106347. 33385926 10.1016/j.envint.2020.106347

[hoad030-B58] Zhao JQ , Hao YY, Gong TT, Wei YF, Zheng G, Du ZD, Zou BJ, Yan S, Liu FH, Gao S et al Phytosterol intake and overall survival in newly diagnosed ovarian cancer patients: an ambispective cohort study. Front Nutr 2022;9:974367. 36091246 10.3389/fnut.2022.974367 PMC9452643

[hoad030-B59] Zhao J-Q , Ma Q-P, Wei Y-F, Zheng G, Zou B-J, Du Z-D, Gao S, Yan S, Qin X, Gong T-T et al Nutrients-Rich Food Index Scores and the Overall Survival of Ovarian Cancer Patients: Results from the Ovarian Cancer Follow-Up Study, a Prospective Cohort Study. Nutrients 2023;15:717. 36771422 10.3390/nu15030717 PMC9920592

[hoad030-B60] Zuccarello D , Ferlin A, Garolla A, Pati MA, Moretti A, Cazzadore C, Francavilla S, Foresta C. A possible association of a human tektin-t gene mutation (A229V) with isolated non-syndromic asthenozoospermia: case report. Hum Reprod 2008;23:996–1001. 18227105 10.1093/humrep/dem400

