# Supplementary material for: Dietary fat and fatty acid consumptions and the odds of asthenozoospermia: a case–control study in China
Source: Hum Reprod Open. 2023 Jul 27;2023(3):hoad030. doi: 10.1093/hropen/hoad030 (PMC10403433; doi:10.1093/hropen/hoad030)
Supplement: hoad030_Supplementary_Figures [file hoad030_supplementary_figures.docx]

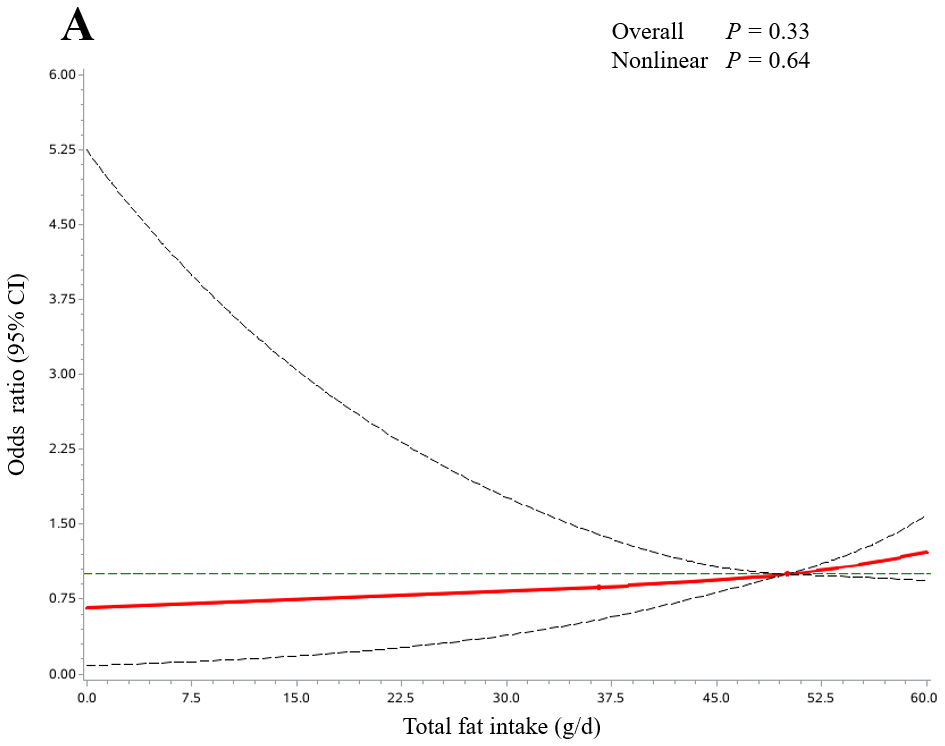

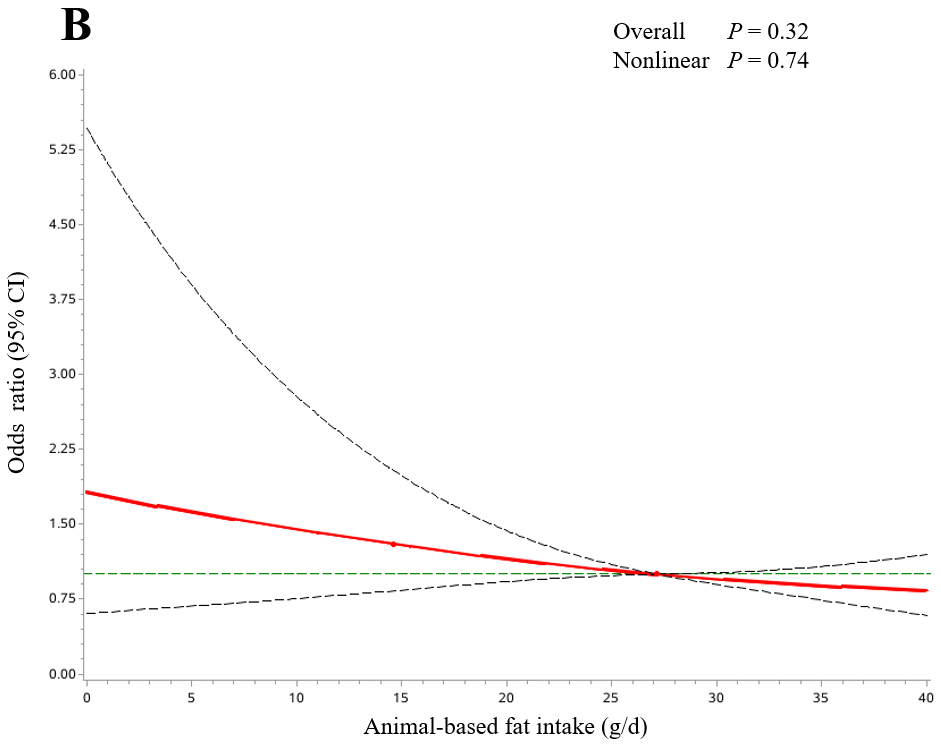

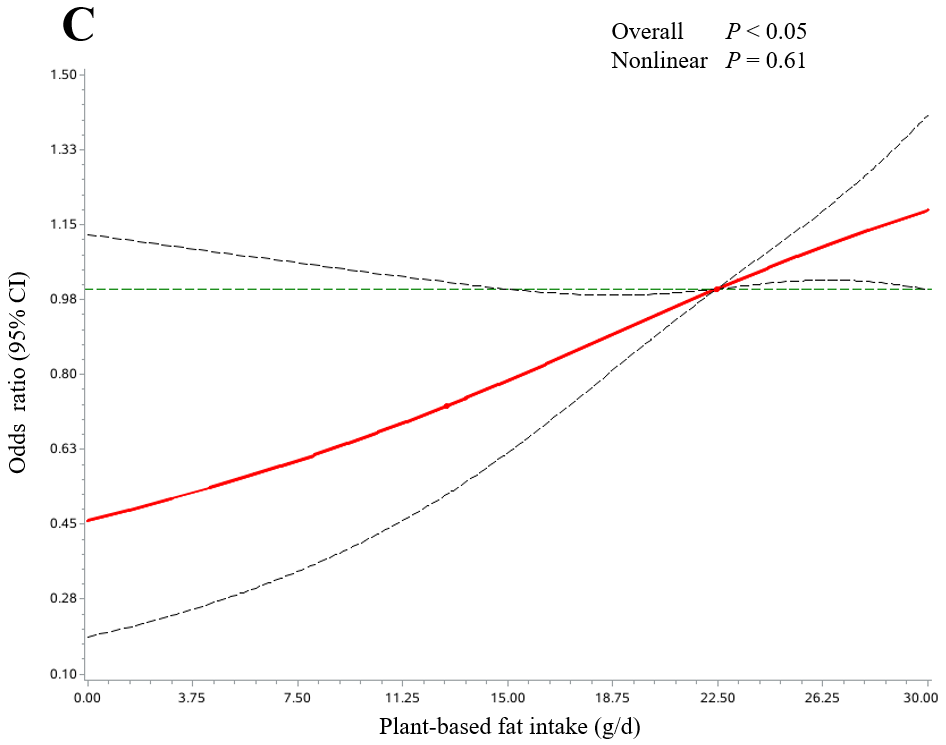


**Supplementary Figure S1.** ORs and 95% CIs of the odds of asthenozoospermia by total fat (A), animal-based fat (B), and plant-based fat (C) intake.

The association was adjusted for age, BMI, alcohol drinking, cigarette smoking, dietary change, household income, education, physical activity, abstinence time, and total energy, total protein, and total carbohydrate intake. The red line and dashed line represent the estimated ORs and their 95% CIs, respectively. Abbreviations: BMI, body mass index; CI, confidence interval; OR, odds ratio.


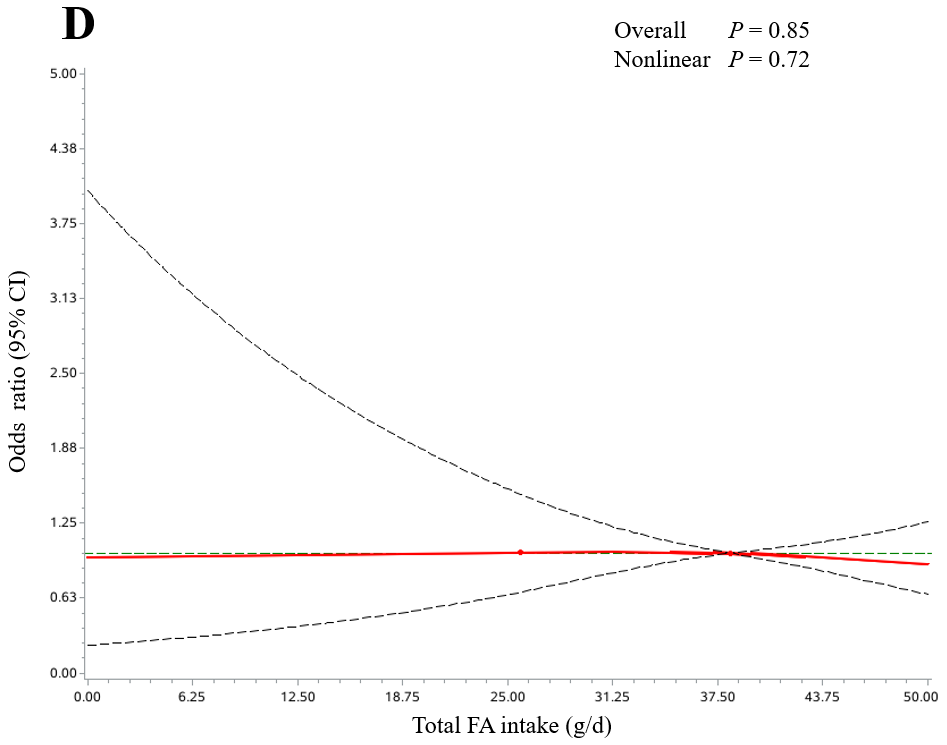

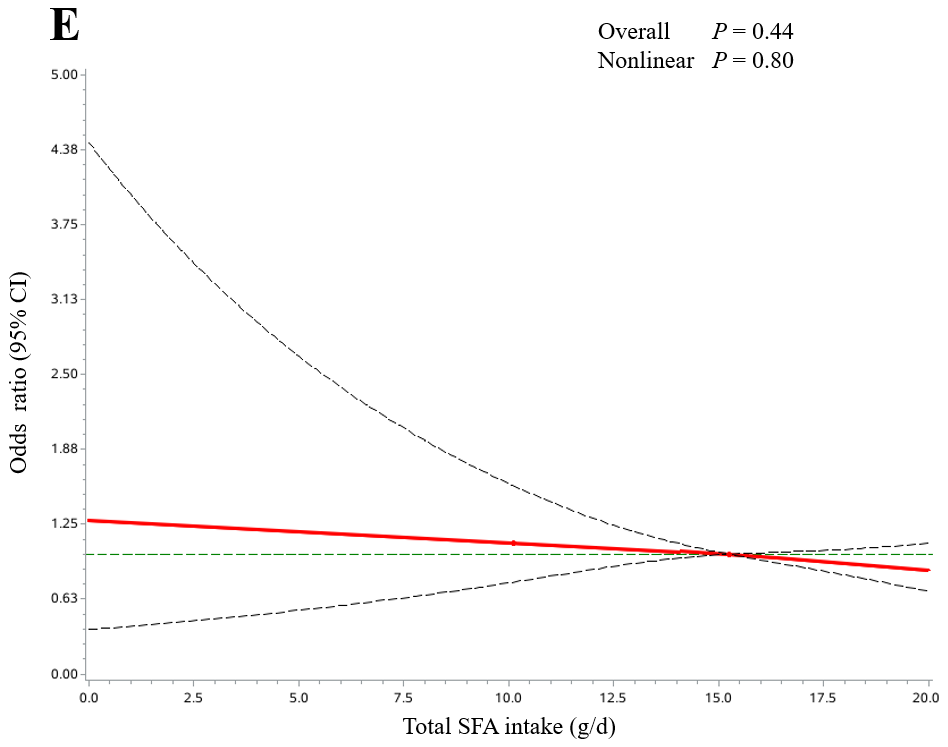

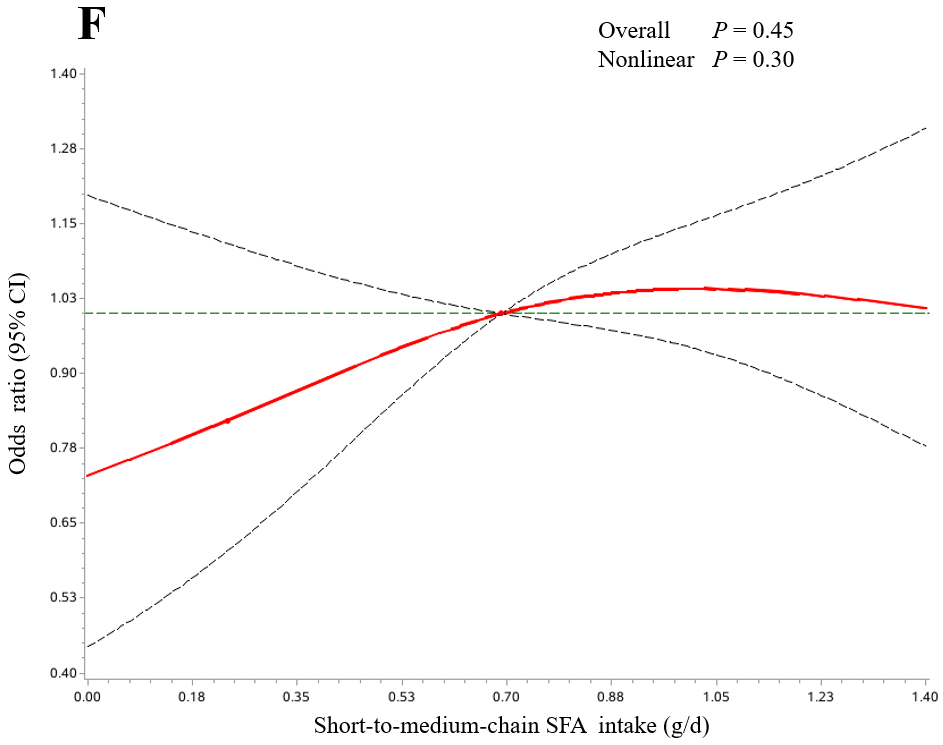


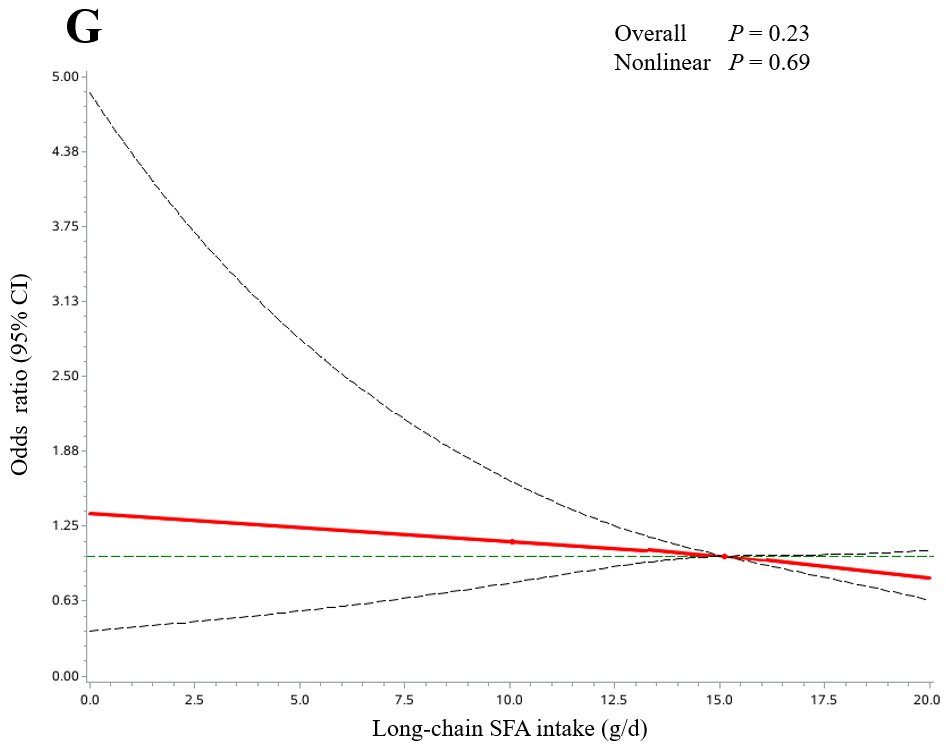


**Supplementary Figure S2.** ORs and 95% CIs of the odds of asthenozoospermia by total FA (D), total SFA (E), short-to-medium-chain SFA (F), and long-chain SFA intake.

The association was adjusted for age, BMI, alcohol drinking, cigarette smoking, dietary change, household income, education, physical activity, abstinence time, and total energy, total protein, and total carbohydrate intake. The red line and dashed line represent the estimated ORs and their 95% CIs, respectively. Abbreviations: BMI, body mass index; CI, confidence interval; FA, fatty acid; OR, odds ratio; SFA, saturated fatty acid.

**
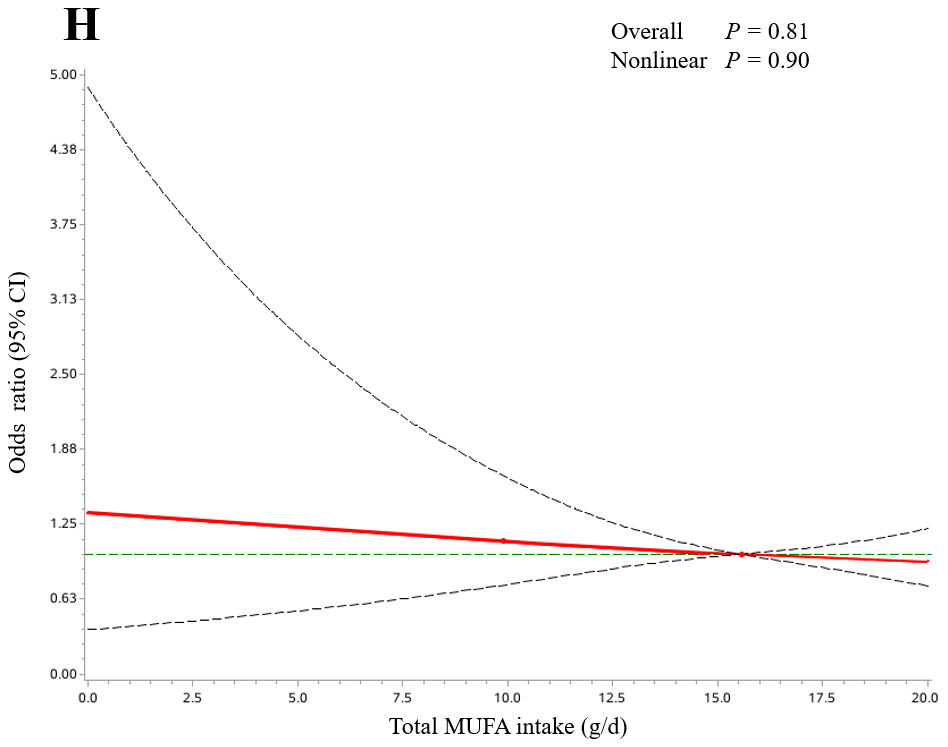

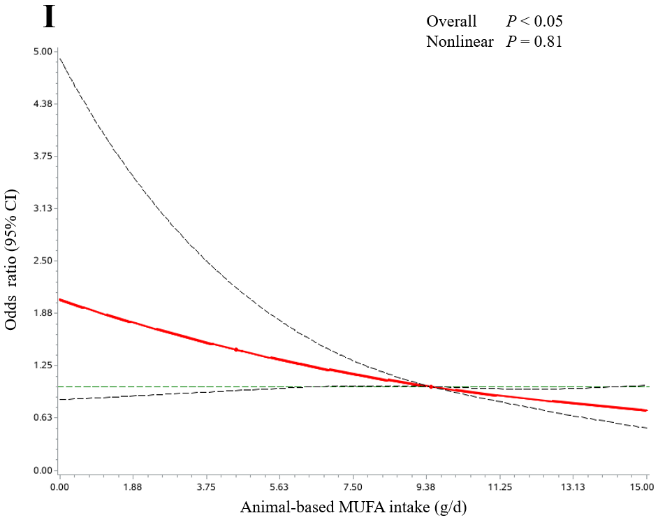
**

**
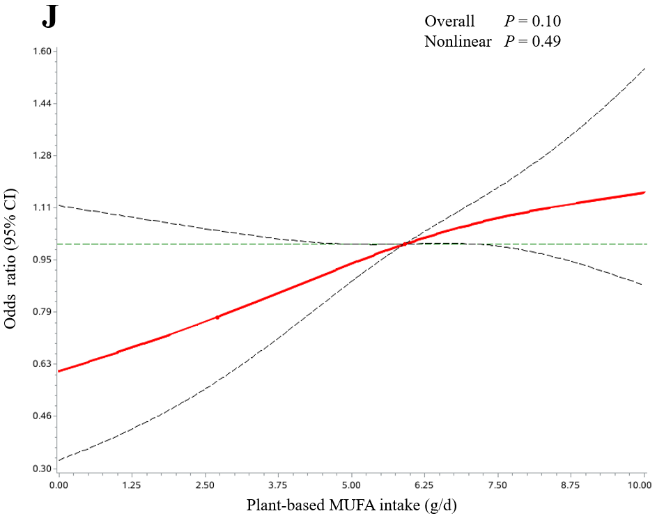
**

**Supplementary Figure S3.** ORs and 95% CIs of the odds of asthenozoospermia by total MUFA (H), animal-based MUFA (I), and plant-based MUFA (J) intake.

The association was adjusted for age, BMI, alcohol drinking, cigarette smoking, dietary change, household income, education, physical activity, abstinence time, and total energy, total protein, and total carbohydrate intake. The red line and dashed line represent the estimated ORs and their 95% CIs, respectively. Abbreviations: BMI, body mass index; CI, confidence interval; MUFA, monounsaturated fatty acid; OR, odds ratio.


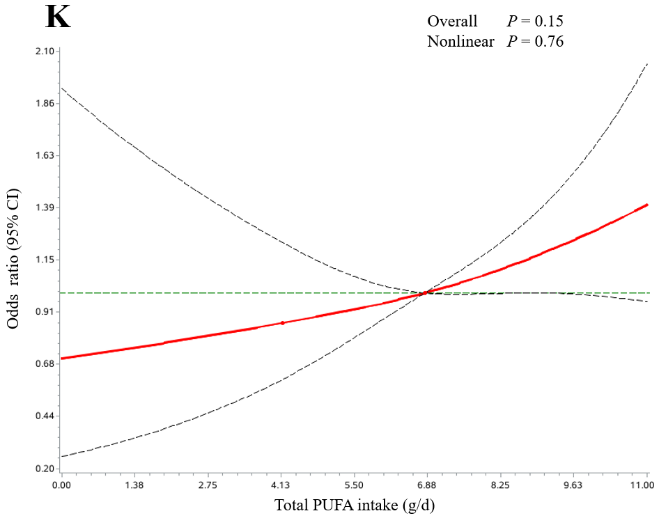

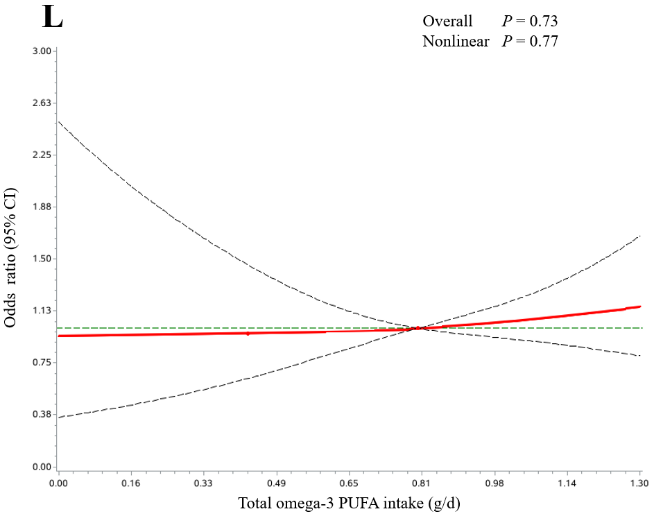


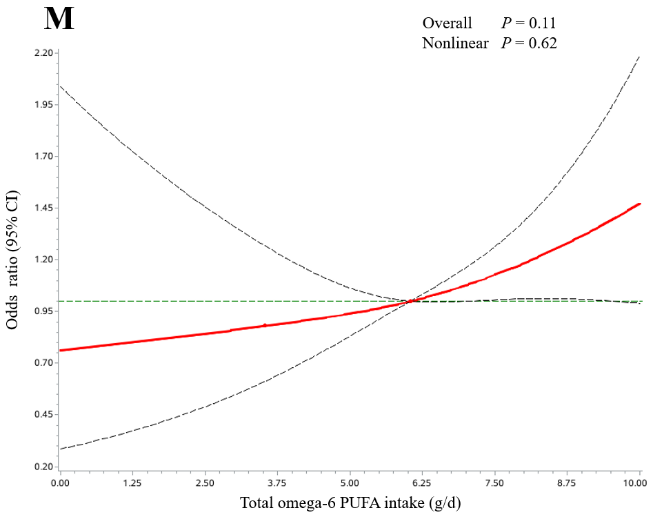
**
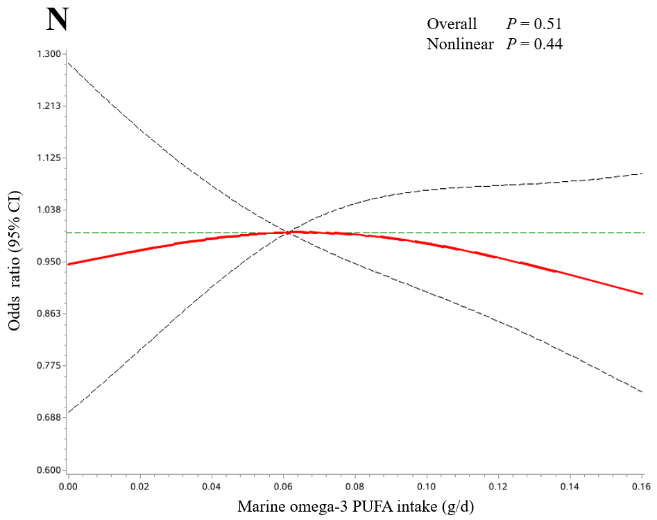

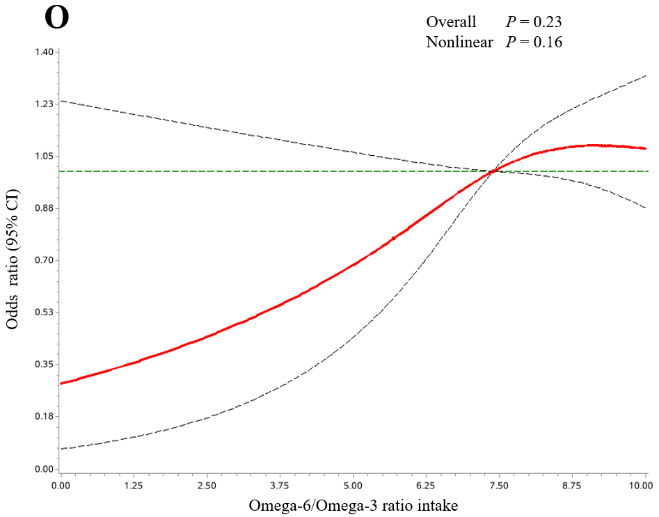
**

**Supplementary Figure S4.** ORs and 95% CIs of the odds of asthenozoospermia by total omega-3 PUFA (L), total omega-6 PUFA (M), marine omega-3 PUFA (N), and omega-6/omega-3 ratio (O) intake.

The association was adjusted for age, BMI, alcohol drinking, cigarette smoking, dietary change, household income, education, physical activity, abstinence time, and total energy, total protein, and total carbohydrate intake. The red line and dashed line represent the estimated ORs and their 95% CIs, respectively. Abbreviations: BMI, body mass index; CI, confidence interval; OR, odds ratio; PUFA, polyunsaturated fatty acid.

**
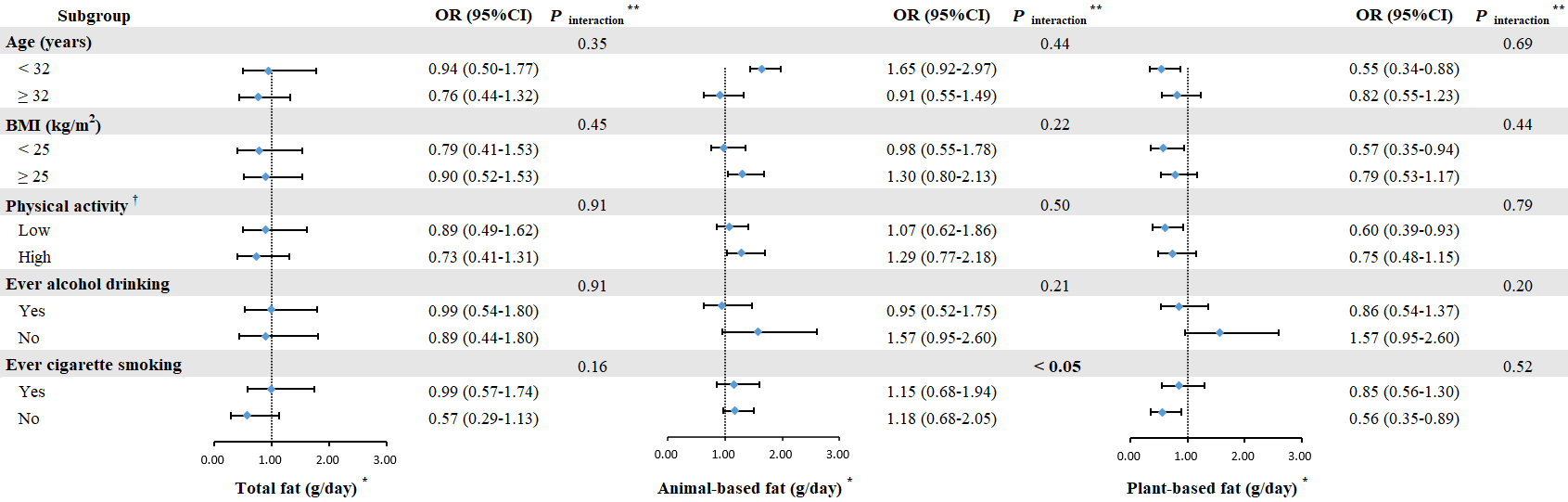
**

**Supplementary Figure S5.** Subgroup analyses for the associations of dietary fat with the odds of asthenozoospermia, stratified by age, BMI, physical activity, alcohol drinking, and cigarette smoking.

^*^ Energy adjustment by residual method.

^**^ Test for interaction based on strata and dietary fat intake.

^†^ Physical activity was categorized by the median of the control groups. MET/hours/week ≤ 127.57 represents low physical activity, while MET/hours/week > 127.57 represents high physical activity.

ORs and 95% CIs were calculated through comparing the highest tertile with the lowest tertile of dietary fat intake, using the unconditional logistic regression model with adjustment for age, BMI, alcohol drinking, cigarette smoking, dietary change, household income, education, physical activity, abstinence time, and total energy, total protein, and total carbohydrate intake.

Abbreviations: BMI, body mass index; CI, confidence interval; OR, odds ratio; Ref, reference; T, tertile.

**
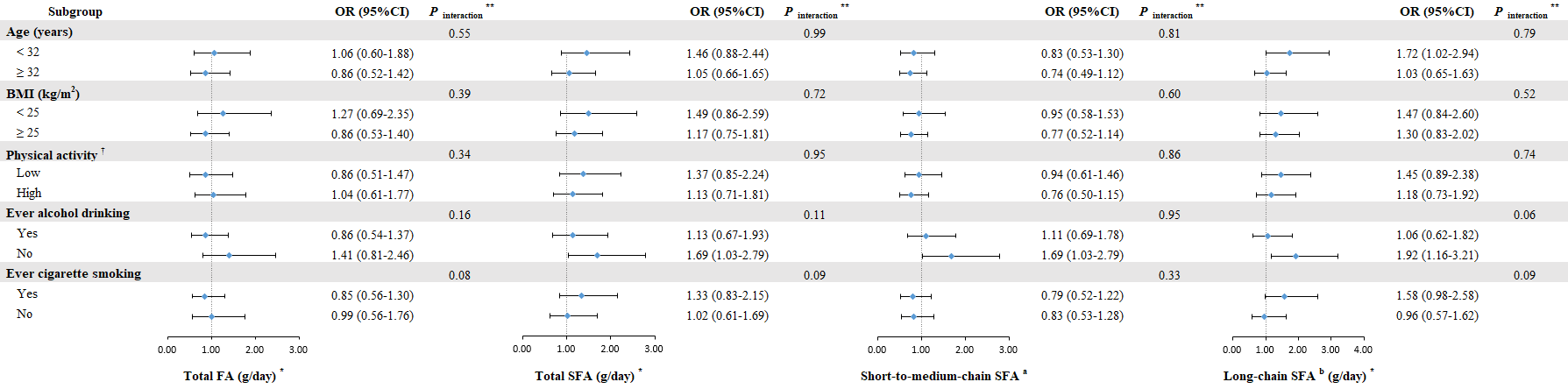
**

**Supplementary Figure S6.** Subgroup analyses for the associations of dietary FA and SFA intake with the odds of asthenozoospermia, stratified by age, BMI, physical activity, alcohol drinking, and cigarette smoking.

^*^ Energy adjustment by residual method.

^**^ Test for interaction based on strata and dietary FA or SFA intake.

^†^ Physical activity was categorized by the median of the control groups. MET/hours/week ≤ 127.57 represents low physical activity, while MET/hours/week > 127.57 represents high physical activity.

^a^ Short-to-medium-chain SFA included saturated butyric (C4), caproic (C6), caprylic (C8), capric (C10), undecanoic (C11), lauric (C12), and tridecanoic (C13) acids.

^b^ Long-chain SFA included saturated myristic (C14), pentadecanoic (C15), palmitic (C16), heptadecanoic (C17), stearic (C18), nonadecanoic (C19), arachidic (C20), behenic (C22), and lignoceric (C24) acids.

ORs and 95% CIs were calculated through comparing the highest tertile with the lowest tertile of dietary FA and SFA intake, using the unconditional logistic regression model with adjustment for age, BMI, alcohol drinking, cigarette smoking, dietary change, household income, education, physical activity, abstinence time, and total energy, total protein, and total carbohydrate intake.

Abbreviations: BMI, body mass index; CI, confidence interval; FA, fatty acid; OR, odds ratio; Ref, reference; SFA, saturated fatty acid; T, tertile.

**
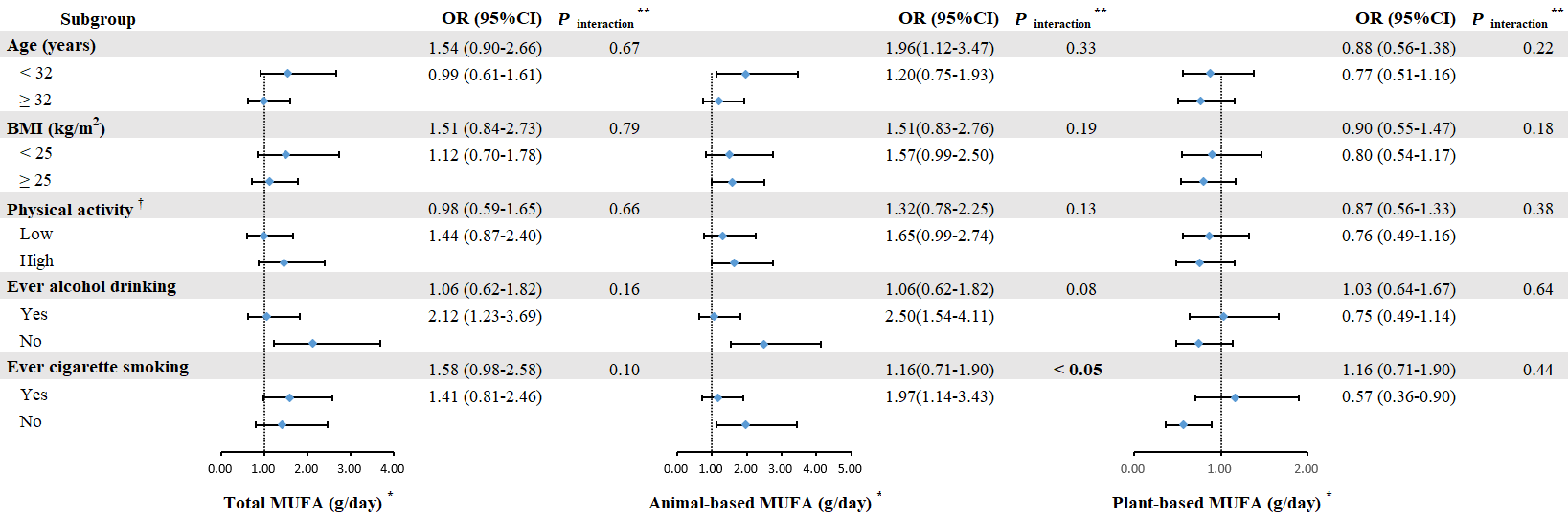
**

**Supplementary Figure S7.** Subgroup analyses for the associations of dietary MUFA intake with the odds of asthenozoospermia, stratified by age, BMI, physical activity, alcohol drinking, and cigarette smoking.

^*^ Energy adjustment by residual method.

^**^ Test for interaction based on strata and dietary MUFA intake.

^†^ Physical activity was categorized by the median of the control groups. MET/hours/week ≤ 127.57 represents low physical activity, while MET/hours/week > 127.57 represents high physical activity.

ORs and 95% CIs were calculated through comparing the highest tertile with the lowest tertile of dietary MUFA intake, using the unconditional logistic regression model with adjustment for age, BMI, alcohol drinking, cigarette smoking, dietary change, household income, education, physical activity, abstinence time, and total energy, total protein, and total carbohydrate intake.

Abbreviations: BMI, body mass index; CI, confidence interval; MUFA, monounsaturated fatty acid; OR, odds ratio; Ref, reference; T, tertile.

**
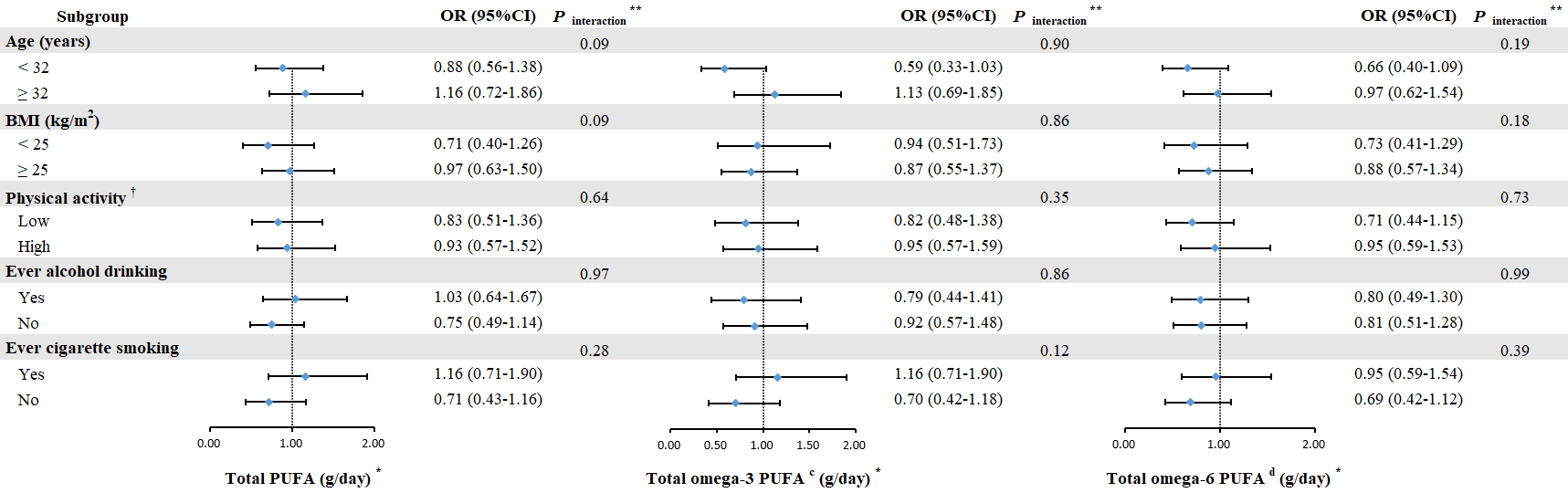
**

**Supplementary Figure S8.** Subgroup analyses for the associations of dietary PUFA intake with the odds of asthenozoospermia, stratified by age, BMI, physical activity, alcohol drinking, and cigarette smoking.

^*^ Energy adjustment by residual method.

^**^ Test for interaction based on strata and dietary PUFA intake.

^†^ Physical activity was categorized by the median of the control groups. MET/hours/week ≤ 127.57 represents low physical activity, while MET/hours/week > 127.57 represents high physical activity.

^c^ Total omega-3 PUFA included alpha-linolenic acid, parinaric acid, docosatrienoic acid, eicosapentaenoic acid (EPA), docosapentaenoic acid (DPA), and docosahexaenoic acid (DHA).

^d^ Total omega-6 PUFA included linoleic acid, eicosadienoic acid, arachidonic acid, and docosatetraenoic acid.

ORs and 95% CIs were calculated through comparing the highest tertile with the lowest tertile of dietary PUFA intake, using the unconditional logistic regression model with adjustment for age, BMI, alcohol drinking, cigarette smoking, dietary change, household income, education, physical activity, abstinence time, and total energy, total protein, and total carbohydrate intake.

Abbreviations: BMI, body mass index; CI, confidence interval; OR, odds ratio; PUFA, polyunsaturated fatty acid; Ref, reference; T, tertile.

**
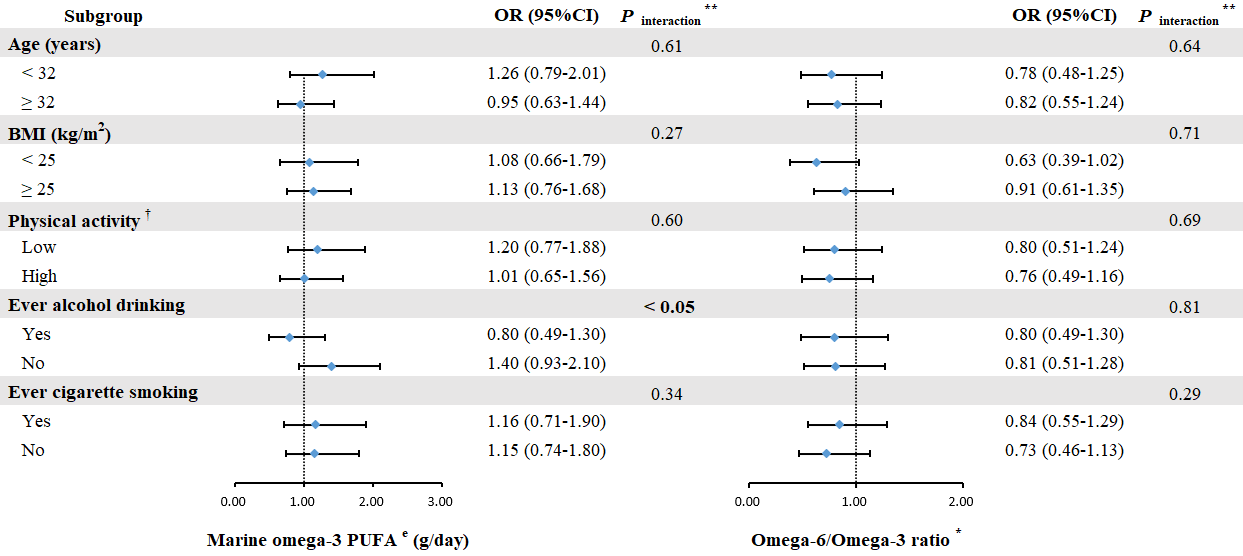
**

**Supplementary Figure S9.** Subgroup analyses for the associations of dietary marine omega-3 PUFA and omega-6/omega-3 PUFA ratio intake with the odds of asthenozoospermia, stratified by age, BMI, physical activity, alcohol drinking, and cigarette smoking.

^*^ Energy adjustment by residual method.

^**^ Test for interaction based on strata and dietary marine omega-3 PUFA or omega-6/omega-3 PUFA ratio intake.

^†^ Physical activity was categorized by the median of the control groups. MET/hours/week ≤ 127.57 represents low physical activity, while MET/hours/week > 127.57 represents high physical activity.

^e^ Marine omega-3 PUFA included eicosapentaenoic acid (EPA), docosapentaenoic acid (DPA), and docosahexaenoic acid (DHA).

ORs and 95% CIs were calculated through comparing the highest tertile with the lowest tertile of dietary marine omega-3 PUFA and omega-6/omega-3 PUFA ratio intake, using the unconditional logistic regression model with adjustment for age, BMI, alcohol drinking, cigarette smoking, dietary change, household income, education, physical activity, abstinence time, and total energy, total protein, and total carbohydrate intake.

Abbreviations: BMI, body mass index; CI, confidence interval; OR, odds ratio; PUFA, polyunsaturated fatty acid; Ref, reference; T, tertile.
