# Supplementary material for: Dietary fat and fatty acid consumptions and the odds of asthenozoospermia: a case–control study in China
Source: Hum Reprod Open. 2023 Jul 27;2023(3):hoad030. doi: 10.1093/hropen/hoad030 (PMC10403433; doi:10.1093/hropen/hoad030)
Supplement: hoad030_Supplementary_Tables [file hoad030_supplementary_tables.docx]

**Supplementary Table S1.** RERI and 95%CI for additive interaction of alcohol drinking and cigarette smoking with dietary fat and fatty acid intake ^*^.

| **Variables** | **Ever alcohol drinking** | | **Ever cigarette smoking** | |
| --- | --- | --- | --- | --- |
|  | Yes | | Yes | |
| **Dietary fat and fatty acids intake** ^†^ | RERI | 95% CI | RERI | 95% CI |
| High total fat (≥ 49.47 g/day) | 0.17 | -0.11, 0.46 | 0.19 | -0.09, 0.48 |
| High animal-based fat (≥ 27.45 g/day) | 0.20 | -0.02, 0.42 | 0.18 | -0.05, 0.41 |
| Low plant-based fat (≤ 21.75 g/day) | 0.09 | -0.15, 0.34 | 0.19 | -0.01, 0.39 |
| High total FA (≥ 38.71 g/day) | 0.24 | 0.05, 0.44 | 0.13 | -0.12, 0.38 |
| High total SFA (≥ 15.37 g/day) | 0.16 | -0.08, 0.40 | 0.21 | -0.02, 0.44 |
| Low short-to-medium-chain SFA ^a^ (≤ 0.67 g/day) | 0.17 | -0.06, 0.40 | -0.08 | -0.43, 0.27 |
| High long-chain SFA ^b^ (≥ 15.32 g/day) | 0.15 | -0.08, 0.38 | 0.20 | -0.03, 0.42 |
| High total MUFA (≥ 15.66 g/day) | 0.19 | -0.04, 0.42 | 0.18 | -0.05, 0.42 |
| High animal-based MUFA (≥ 9.66 g/day) | 0.27 | 0.09, 0.46 | 0.23 | 0.03, 0.44 |
| Low plant-based MUFA (≤ 5.86 g/day) | -0.01 | -0.33, 0.32 | 0.02 | -0.29, 0.33 |
| Low total PUFA (≤ 6.81 g/day) | -0.02 | -0.35, 0.30 | -0.17 | -0.58, 0.23 |
| High total omega-3 PUFA ^c^ (≥ 0.81 g/day) | 0.19 | -0.05, 0.43 | 0.14 | -0.13, 0.40 |
| Low total omega-6 PUFA ^d^ (≤ 6.01 g/day) | -0.01 | -0.32, 0.30 | -0.21 | -0.63, 0.20 |
| High marine omega-3 PUFA ^e^ (≥ 0.06 g/day) | 0.18 | -0.06, 0.42 | 0.13 | -0.14, 0.39 |
| Low omega-6/omega-3 ratio (≤ 7.32) | 0.10 | -0.16, 0.36 | 0.04 | -0.25, 0.32 |

Abbreviations: CI, confidence interval; FA, fatty acid; MUFA, monounsaturated fatty acid; PUFA, polyunsaturated fatty acid; RERI, relative excess risk due to interaction; SFA, saturated fatty acid.

^*^ RERI and 95% CI were calculated with the use of the unconditional logistic regression model with adjustment for age (continuous, years), BMI (continuous, kg/m^2^), alcohol drinking (yes or no), cigarette smoking (yes or no), dietary change (yes or no), household income (< 50, 50 to <100, or ≥ 100, thousand yuan), education (junior secondary or below, senior high school/technical secondary school, and junior college/university or above), physical activity (continuous, MET/hours/week), abstinence time (continuous, days), and total energy (continuous, kcal/day), total protein (continuous, g/day), and total carbohydrate (continuous, g/day) intake.

^†^ Adjusted for energy by the residual method.

^a^ Short-to-medium-chain SFA included saturated butyric (C4), caproic (C6), caprylic (C8), capric (C10), undecanoic (C11), lauric (C12), tridecanoic (C13) acids.

^b^ Long-chain SFA included saturated myristic (C14), pentadecanoic (C15), palmitic (C16), heptadecanoic (C17), stearic (C18), nonadecanoic (C19), arachidic (C20), behenic (C22), lignoceric (C24) acids.

^c^ Total omega-3 PUFA included alpha-linolenic acid, parinaric acid, docosatrienoic acid, eicosapentaenoic acid (EPA), docosapentaenoic acid (DPA), and docosahexaenoic acid (DHA).

^d^ Total omega-6 PUFA included linoleic acid, eicosadienoic acid, arachidonic acid, and docosatetraenoic acid.

^e^ Marine omega-3 PUFA included eicosapentaenoic acid (EPA), docosapentaenoic acid (DPA), and docosahexaenoic acid (DHA).

**Supplementary Table S2.** RERI and 95%CI for additive interaction of age, BMI, and physical activity with dietary fat and fatty acid intake ^*^.

| **Variables** | **Age** | | **BMI** | | **Physical activity** ^**^ | |
| --- | --- | --- | --- | --- | --- | --- |
|  | Old age ( ≥ 32 years) | | High BMI (≥ 25 kg/m^2^) | | Low physical activity | |
| **Dietary fat and fatty acid intake** ^†^ | RERI | 95% CI | RERI | 95% CI | RERI | 95% CI |
| High total fat (≥ 49.47 g/day) | -0.13 | -0.52, 0.26 | 0.06 | -0.32, 0.43 | -0.21 | -0.61, 0.20 |
| High animal-based fat (≥ 27.45 g/day) | -0.14 | -0.42, 0.13 | 0.21 | -0.09, 0.52 | -0.09 | -0.36, 0.18 |
| Low plant-based fat (≤ 21.75 g/day) | -0.02 | -0.27, 0.23 | 0.03 | -0.35, 0.42 | 0.06 | -0.16, 0.29 |
| High total FA (≥ 38.71 g/day) | -0.17 | -0.45, 0.10 | -0.11 | -0.47, 0.25 | -0.08 | -0.34, 0.17 |
| High total SFA (≥ 15.37 g/day) | -0.17 | -0.46, 0.12 | 0.06 | -0.29, 0.40 | -0.13 | -0.41, 0.15 |
| Low short-to-medium-chain SFA ^a^ (≤ 0.67 g/day) | 0.11 | -0.16, 0.39 | -0.18 | -0.59, 0.24 | -0.04 | -0.30, 0.22 |
| High long-chain SFA ^b^ (≥ 15.32 g/day) | -0.13 | -0.40, 0.14 | 0.01 | -0.34, 0.35 | -0.04 | -0.30, 0.22 |
| High total MUFA (≥ 15.66 g/day) | -0.16 | -0.46, 0.14 | -0.22 | -0.60, 0.17 | -0.17 | -0.45, 0.11 |
| High animal-based MUFA (≥ 9.66 g/day) | -0.22 | -0.50, 0.06 | 0.08 | -0.26, 0.42 | -0.18 | -0.45, 0.09 |
| Low plant-based MUFA (≤ 5.86 g/day) | -0.05 | -0.35, 0.24 | -0.05 | -0.43, 0.33 | 0.01 | -0.27, 0.29 |
| Low total PUFA (≤ 6.81 g/day) | -0.06 | -0.36, 0.24 | -0.08 | -0.27, 0.43 | 0.18 | -0.09, 0.45 |
| High total omega-3 PUFA ^c^ (≥ 0.81 g/day) | -0.03 | -0.34, 0.28 | 0.14 | -0.20, 0.47 | -0.28 | -0.58, 0.02 |
| Low total omega-6 PUFA ^d^ (≤ 6.01 g/day) | -0.07 | -0.35, 0.21 | 0.09 | -0.27, 0.44 | 0.18 | -0.08, 0.44 |
| High marine omega-3 PUFA ^e^ (≥ 0.06 g/day) | -0.06 | -0.38, 0.25 | 0.28 | -0.01, 0.57 | -0.09 | -0.40, 0.21 |
| Low omega-6/omega-3 ratio (≤ 7.32) | -0.10 | -0.37, 0.17 | -0.17 | -0.58, 0.24 | -0.04 | -0.30, 0.22 |

Abbreviations: BMI, body mass index; CI, confidence interval; FA, fatty acid; MUFA, monounsaturated fatty acid; PUFA, polyunsaturated fatty acid; RERI, relative excess risk due to interaction; SFA, saturated fatty acid.

^*^ RERI and 95% CI were calculated with the use of the unconditional logistic regression model with adjustment for age (continuous, years), BMI (continuous, kg/m^2^), alcohol drinking (yes or no), cigarette smoking (yes or no), dietary change (yes or no), household income (< 50, 50 to <100, or ≥ 100, thousand yuan), education (junior secondary or below, senior high school/technical secondary school, and junior college/university or above), physical activity (continuous, MET/hours/week), abstinence time (continuous, days), and total energy (continuous, kcal/day), total protein (continuous, g/day), and total carbohydrate (continuous, g/day) intake.

^**^ Physical activity was categorized by the median of the control groups. MET/hours/week ≤ 127.57 represents low physical activity, while MET/hours/week > 127.57 represents high physical activity.

^†^ Adjusted for energy by the residual method.

^a^ Short-to-medium-chain SFA included saturated butyric (C4), caproic (C6), caprylic (C8), capric (C10), undecanoic (C11), lauric (C12), tridecanoic (C13) acids.

^b^ Long-chain SFA included saturated myristic (C14), pentadecanoic (C15), palmitic (C16), heptadecanoic (C17), stearic (C18), nonadecanoic (C19), arachidic (C20), behenic (C22), lignoceric (C24) acids.

^c^ Total omega-3 PUFA included alpha-linolenic acid, parinaric acid, docosatrienoic acid, eicosapentaenoic acid (EPA), docosapentaenoic acid (DPA), and docosahexaenoic acid (DHA).

^d^ Total omega-6 PUFA included linoleic acid, eicosadienoic acid, arachidonic acid, and docosatetraenoic acid.

^e^ Marine omega-3 PUFA included eicosapentaenoic acid (EPA), docosapentaenoic acid (DPA), and docosahexaenoic acid (DHA).

**Supplementary Table S3.** Sensitivity analyses: adjusted ORs and 95% CIs for the associations of dietary fat and fatty acid intake with the odds of asthenozoospermia using nutrient-density method.

| **Characteristics** | | **Multivariable-adjusted models** | | |
| --- | --- | --- | --- | --- |
|  |  | **Model 1** | **Model 2** | **Model 3** |
| **Total fat (g/1000kcal)** ^†^ | T1 (< 25.39) | 1.00 (Ref) | 1.00 (Ref) | 1.00 (Ref) |
|  | T2 ( 25.39 to < 29.76) | 1.22 (0.90-1.65) | 1.31 (0.97-1.78) | 1.10 (0.79-1.55) |
|  | T3 (≥29.76) | 1.02 (0.76-1.36) | 1.10 (0.81-1.48) | 0.76 (0.50-1.17) |
|  | Continuous (per SD increment) | 1.01 (0.90-1.15) | 1.04 (0.92-1.18) | 0.83 (0.67-1.03) |
|  | *P* for trend ^*^ | 0.98 | 0.63 | 0.19 |
| **Animal-based fat (g/1000kcal)** ^†^ | T1 (<13.44) | 1.00 (Ref) | 1.00 (Ref) | 1.00 (Ref) |
|  | T2 (13.44 to < 16.95) | 1.01 (0.76-1.34) | 0.99 (0.74-1.32) | 0.96 (0.70-1.30) |
|  | T3 (≥ 16.95) | 1.38 (1.03-1.85) | 1.42 (1.05-1.91) | 1.32 (0.89-1.95) |
|  | Continuous (per SD increment) | 1.13 (1.01-1.28) | 1.14 (1.01-1.29) | 1.11 (0.93-1.34) |
|  | *P* for trend ^*^ | < 0.05 | < 0.05 | 0.20 |
| **Plant-based fat (g/1000kcal)** ^†^ | T1 (< 10.44) | 1.00 (Ref) | 1.00 (Ref) | 1.00 (Ref) |
|  | T2 (10.44 to < 13.46) | 0.76 (0.56-1.03) | 0.78 (0.58-1.06) | 0.77 (0.57-1.05) |
|  | T3 (≥ 13.46) | 0.66 (0.48-0.91) | 0.70 (0.50-0.96) | 0.64 (0.46-0.89) |
|  | Continuous (per SD increment) | 0.85 (0.74-0.96) | 0.87 (0.76-0.99) | 0.83 (0.72-0.95) |
|  | *P* for trend ^*^ | < 0.05 | < 0.05 | < 0.05 |
| **Total FA (g/1000kcal)** ^†^ | T1 (< 19.26) | 1.00 (Ref) | 1.00 (Ref) | 1.00 (Ref) |
|  | T2 (19.26 to < 22.97) | 1.23 (0.92-1.65) | 1.27 (0.94-1.71) | 1.17 (0.85-1.61) |
|  | T3 (≥ 22.97) | 1.18 (0.88-1.58) | 1.24 (0.92-1.67) | 1.04 (0.70-1.53) |
|  | Continuous (per SD increment) | 1.09 (0.97-1.23) | 1.11 (0.99-1.26) | 1.04 (0.86-1.24) |
|  | *P* for trend ^*^ | 0.29 | 0.16 | 0.88 |
| **Total SFA (g/1000kcal)** ^†^ | T1 (< 7.51) | 1.00 (Ref) | 1.00 (Ref) | 1.00 (Ref) |
|  | T2 (7.51 to < 9.22) | 1.24 (0.93-1.65) | 1.29 (0.96-1.73) | 1.21 (0.88-1.66) |
|  | T3 (≥ 9.22) | 1.28 (0.96-1.72) | 1.37 (1.02-1.85) | 1.24 (0.87-1.76) |
|  | Continuous (per SD increment) | 1.11 (0.98-1.25) | 1.13 (1.00-1.28) | 1.08 (0.93-1.26) |
|  | *P* for trend ^*^ | 0.10 | < 0.05 | 0.26 |
| **Short-to-medium-chain SFA ^a^ (g/1000kcal)** ^†^ | T1 (< 0.28) | 1.00 (Ref) | 1.00 (Ref) | 1.00 (Ref) |
|  | T2 (0.28 to < 0.47) | 0.81 (0.60-1.10) | 0.85 (0.63-1.16) | 0.84 (0.62-1.14) |
|  | T3 (≥ 0.47) | 0.72 (0.53-0.97) | 0.77 (0.57-1.05) | 0.74 (0.54-1.01) |
|  | Continuous (per SD increment) | 0.93 (0.82-1.05) | 0.96 (0.84-1.08) | 0.94 (0.83-1.07) |
|  | *P* for trend ^*^ | < 0.05 | 0.12 | 0.07 |
| **Long-chain SFA ^b^ (g/1000kcal)** ^†^ | T1 (< 7.47) | 1.00 (Ref) | 1.00 (Ref) | 1.00 (Ref) |
|  | T2 (7.47 to < 9.11) | 1.25 (0.94-1.67) | 1.28 (0.96-1.72) | 1.21 (0.89-1.66) |
|  | T3 (≥ 9.11) | 1.30 (0.97-1.74) | 1.37 (1.02-1.84) | 1.23 (0.86-1.76) |
|  | Continuous (per SD increment) | 1.14 (1.01-1.28) | 1.16 (1.03-1.31) | 1.12 (0.96-1.31) |
|  | *P* for trend ^*^ | 0.08 | < 0.05 | 0.26 |
| **Total MUFA (g/1000kcal)** ^†^ | T1 (< 7.85) | 1.00 (Ref) | 1.00 (Ref) | 1.00 (Ref) |
|  | T2 ( 7.85 to < 9.40) | 1.21 (0.91-1.62) | 1.24 (0.93-1.67) | 1.17 (0.86-1.61) |
|  | T3 (≥ 9.40) | 1.32 (0.99-1.77) | 1.35 (1.01-1.82) | 1.20 (0.83-1.75) |
|  | Continuous (per SD increment) | 1.11 (0.98-1.25) | 1.12 (0.99-1.27) | 1.06 (0.89-1.25) |
|  | *P* for trend ^*^ | 0.06 | < 0.05 | 0.34 |
| **Animal-based MUFA (g/1000kcal)** ^†^ | T1 (< 4.52) | 1.00 (Ref) | 1.00 (Ref) | 1.00 (Ref) |
|  | T2 (4.52 to < 6.09) | 1.09 (0.82-1.44) | 1.08 (0.81-1.43) | 1.05 (0.77-1.41) |
|  | T3 (≥ 6.09) | 1.52 (1.14-2.05) | 1.52 (1.13-2.04) | 1.44 (1.00-2.08) |
|  | Continuous (per SD increment) | 1.20 (1.07-1.36) | 1.20 (1.07-1.36) | 1.21 (1.03-1.43) |
|  | *P* for trend ^*^ | < 0.05 | < 0.05 | 0.05 |
| **Plant-based MUFA (g/1000kcal)** ^†^ | T1 (< 2.59) | 1.00 (Ref) | 1.00 (Ref) | 1.00 (Ref) |
|  | T2 (2.59 to < 3.61) | 0.85 (0.63-1.14) | 0.88 (0.65-1.18) | 0.86 (0.64-1.17) |
|  | T3 (≥ 3.61) | 0.75 (0.55-1.02) | 0.79 (0.58-1.08) | 0.75 (0.55-1.02) |
|  | Continuous (per SD increment) | 0.89 (0.78-1.01) | 0.91 (0.80-1.03) | 0.88 (0.77-1.01) |
|  | *P* for trend ^*^ | 0.07 | 0.14 | 0.07 |
| **Total PUFA (g/1000kcal)** ^†^ | T1 (< 3.26) | 1.00 (Ref) | 1.00 (Ref) | 1.00 (Ref) |
|  | T2 (3.26 to < 4.19) | 1.27 (0.95-1.72) | 1.33 (0.99-1.81) | 1.21 (0.89-1.66) |
|  | T3 (≥ 4.19) | 0.97 (0.73-1.30) | 1.01 (0.76-1.36) | 0.82 (0.57-1.17) |
|  | Continuous (per SD increment) | 0.98 (0.87-1.11) | 0.99 (0.88-1.12) | 0.87 (0.74-1.03) |
|  | *P* for trend ^*^ | 0.61 | 0.79 | 0.15 |
| **Total omega-3 PUFA ^c^ (g/1000kcal)** ^†^ | T1 (< 0.38) | 1.00 (Ref) | 1.00 (Ref) | 1.00 (Ref) |
|  | T2 (0.38 to < 0.50) | 1.20 (0.90-1.61) | 1.24 (0.92-1.66) | 1.13 (0.82-1.55) |
|  | T3 (≥ 0.50) | 1.06 (0.80-1.42) | 1.09 (0.82-1.46) | 0.90 (0.62-1.32) |
|  | Continuous (per SD increment) | 1.04 (0.92-1.17) | 1.05 (0.93-1.19) | 0.95 (0.79-1.14) |
|  | *P* for trend ^*^ | 0.78 | 0.66 | 0.49 |
| **Total omega-6 PUFA ^d^ (g/1000kcal)** ^†^ | T1 (< 2.87) | 1.00 (Ref) | 1.00 (Ref) | 1.00 (Ref) |
|  | T2 (2.87 to < 3.70) | 1.25 (0.93-1.69) | 1.31 (0.97-1.77) | 1.20 (0.88-1.64) |
|  | T3 (≥ 3.70) | 0.96 (0.72-1.28) | 1.00 (0.74-1.34) | 0.82 (0.58-1.15) |
|  | Continuous (per SD increment) | 0.97 (0.86-1.09) | 0.98 (0.87-1.11) | 0.87 (0.74-1.02) |
|  | *P* for trend ^*^ | 0.54 | 0.71 | 0.13 |
| **Marine omega-3 PUFA ^e^ (g/1000kcal)** ^†^ | T1 (< 0.02) | 1.00 (Ref) | 1.00 (Ref) | 1.00 (Ref) |
|  | T2 (0.02 to < 0.05) | 1.21 (0.90-1.63) | 1.17 (0.87-1.58) | 1.11 (0.82-1.51) |
|  | T3 (≥ 0.05) | 1.22 (0.91-1.63) | 1.18 (0.88-1.58) | 1.10 (0.81-1.51) |
|  | Continuous (per SD increment) | 1.07 (0.95-1.22) | 1.06 (0.94-1.21) | 1.04 (0.91-1.18) |
|  | *P* for trend ^*^ | 0.21 | 0.32 | 0.58 |
| **Omega-6/Omega-3 ratio** ^†^ | T1 (< 3.72) | 1.00 (Ref) | 1.00 (Ref) | 1.00 (Ref) |
|  | T2 (3.72 to < 5.09) | 1.01 (0.73-1.42) | 1.03 (0.73-1.45) | 1.06 (0.75-1.50) |
|  | T3 (≥ 5.09) | 1.04 (0.68-1.58) | 1.10 (0.72-1.68) | 1.19 (0.77-1.86) |
|  | Continuous (per SD increment) | 0.93 (0.78-1.10) | 0.94 (0.79-1.12) | 0.98 (0.82-1.18) |
|  | *P* for trend ^*^ | 0.85 | 0.65 | 0.41 |

Abbreviations: BMI, body mass index; CI, confidence interval; FA, fatty acid; MUFA, monounsaturated fatty acid; OR, odds ratio; PUFA, polyunsaturated fatty acid; Ref, reference; SD, standard deviation; SFA, saturated fatty acid; T, tertile.

The standard deviation for the listed fat and fatty acid are 5.26 g/day, 4.52 g/day, 3.86 g/day, 4.67 g/day, 2.05 g/day, 0.23 g/day, 2.01 g/day, 2.06 g/day, 1.87 g/day, 1.31 g/day, 1.20 g/day, 0.16 g/day, 1.07 g/day, 0.04 g/day, and 1.86 respectively.

Model 1: adjusted for age (continuous, years) and total energy intake (continuous, kcal/day).

Model 2: same as model 1 and further adjusted for BMI (continuous, kg/m^2^), alcohol drinking (yes or no), cigarette smoking (yes or no), dietary change (yes or no), household income (< 50, 50 to <100, or ≥ 100 RMB, thousand yuan), education (junior secondary or below, senior high school/technical secondary school, and junior college/university or above), physical activity (continuous, MET/hours/week), and abstinence time (continuous, days).

Model 3: same as model 2 and further adjusted for total protein (continuous, g/day) and total carbohydrate (continuous, g/day) intake.

^†^ Energy adjustment by nutrient-density method.

^*^ Test for trend based on variables containing the median value for each tertile.

^a^ Short-to-medium-chain SFA included saturated butyric (C4), caproic (C6), caprylic (C8), capric (C10), undecanoic (C11), lauric (C12), tridecanoic (C13) acids.

^b^ Long-chain SFA included saturated myristic (C14), pentadecanoic (C15), palmitic (C16), heptadecanoic (C17), stearic (C18), nonadecanoic (C19), arachidic (C20), behenic (C22), lignoceric (C24) acids.

^c^ Total omega-3 PUFA included alpha-linolenic acid, parinaric acid, docosatrienoic acid, eicosapentaenoic acid (EPA), docosapentaenoic acid (DPA), and docosahexaenoic acid (DHA).

^d^ Total omega-6 PUFA included linoleic acid, eicosadienoic acid, arachidonic acid, and docosatetraenoic acid.

^e^ Marine omega-3 PUFA included eicosapentaenoic acid (EPA), docosapentaenoic acid (DPA), and docosahexaenoic acid (DHA).

**Supplementary Table S4.** Sensitivity analyses: adjusted ORs and 95% CIs for the association of dietary fat and fatty acid intake with the odds of asthenozoospermia among the participants without dietary change.

| **Characteristics** | | **Multivariable-adjusted models** | | |
| --- | --- | --- | --- | --- |
|  |  | **Model 1** | **Model 2** | **Model 3** |
| **Total fat (g/day)** ^†^ | T1 (< 46.45) | 1.00 (Ref) | 1.00 (Ref) | 1.00 (Ref) |
|  | T2 (46.45 to < 53.17) | 1.10 (0.79-1.54) | 1.15 (0.82-1.61) | 0.98 (0.68-1.41) |
|  | T3 (≥ 53.17) | 0.99 (0.72-1.37) | 1.05 (0.75-1.46) | 0.71 (0.44-1.12) |
|  | Continuous (per SD increment) | 1.03 (0.90-1.18) | 1.05 (0.92-1.21) | 0.89 (0.72-1.11) |
|  | *P* for trend ^*^ | 0.95 | 0.81 | 0.14 |
| **Animal-based fat (g/day)** ^†^ | T1 (< 24.19) | 1.00 (Ref) | 1.00 (Ref) | 1.00 (Ref) |
|  | T2 ( 24.19 to < 30.15) | 0.91 (0.66-1.26) | 0.89 (0.64-1.24) | 0.83 (0.59-1.18) |
|  | T3 (≥ 30.15) | 1.20 (0.86-1.67) | 1.22 (0.88-1.71) | 1.05 (0.69-1.60) |
|  | Continuous (per SD increment) | 1.12 (0.98-1.28) | 1.12 (0.98-1.29) | 1.08 (0.89-1.30) |
|  | *P* for trend ^*^ | 0.28 | 0.24 | 0.83 |
| **Plant-based fat (g/day)** ^†^ | T1 (< 19.84) | 1.00 (Ref) | 1.00 (Ref) | 1.00 (Ref) |
|  | T2 (19.84 to < 24.59) | 0.97 (0.69-1.36) | 1.04 (0.74-1.48) | 1.05 (0.74-1.48) |
|  | T3 (≥ 24.59) | 0.78 (0.56-1.07) | 0.82 (0.59-1.14) | 0.75 (0.53-1.06) |
|  | Continuous (per SD increment) | 0.90 (0.79-1.03) | 0.92 (0.80-1.05) | 0.88 (0.76-1.02) |
|  | *P* for trend ^*^ | 0.10 | 0.19 | 0.07 |
| **Total FA (g/day)** ^†^ | T1 (< 35.39) | 1.00 (Ref) | 1.00 (Ref) | 1.00 (Ref) |
|  | T2 (35.39 to < 41.18) | 1.06 (0.76-1.47) | 1.06 (0.76-1.48) | 0.96 (0.67-1.36) |
|  | T3 (≥ 41.18) | 1.10 (0.80-1.52) | 1.14 (0.82-1.59) | 0.90 (0.59-1.37) |
|  | Continuous (per SD increment) | 1.11 (0.97-1.27) | 1.13 (0.99-1.30) | 1.07 (0.89-1.30) |
|  | *P* for trend ^*^ | 0.57 | 0.43 | 0.62 |
| **Total SFA (g/day)** ^†^ | T1 (< 13.93) | 1.00 (Ref) | 1.00 (Ref) | 1.00 (Ref) |
|  | T2 ( 13.93 to < 16.59) | 1.22 (0.88-1.69) | 1.25 (0.90-1.75) | 1.19 (0.84-1.67) |
|  | T3 (≥ 16.59) | 1.34 (0.97-1.86) | 1.43 (1.03-2.00) | 1.28 (0.88-1.87) |
|  | Continuous (per SD increment) | 1.15 (1.00-1.31) | 1.18 (1.03-1.35) | 1.13 (0.96-1.33) |
|  | *P* for trend ^*^ | 0.07 | < 0.05 | 0.20 |
| **Short-to-medium-chain SFA ^a^ (g/ day)** ^†^ | T1 (< 0.53) | 1.00 (Ref) | 1.00 (Ref) | 1.00 (Ref) |
|  | T2 (0.53 to < 0.83) | 0.82 (0.59-1.14) | 0.87 (0.62-1.23) | 0.88 (0.62-1.23) |
|  | T3 (≥ 0.83) | 0.88 (0.63-1.22) | 0.94 (0.67-1.31) | 0.91 (0.64-1.28) |
|  | Continuous (per SD increment) | 0.98 (0.85-1.12) | 1.00 (0.87-1.15) | 0.99 (0.87-1.14) |
|  | *P* for trend ^*^ | 0.52 | 0.79 | 0.64 |
| **Long-chain SFA ^b^ (g/day)** ^†^ | T1 (<13.87) | 1.00 (Ref) | 1.00 (Ref) | 1.00 (Ref) |
|  | T2 (13.87 to < 16.38) | 1.25 (0.90-1.72) | 1.29 (0.92-1.79) | 1.23 (0.87-1.74) |
|  | T3 (≥ 16.38) | 1.39 (1.01-1.93) | 1.47 (1.06-2.05) | 1.33 (0.91-1.96) |
|  | Continuous (per SD increment) | 1.17 (1.02-1.34) | 1.20 (1.04-1.38) | 1.16 (0.98-1.37) |
|  | *P* for trend ^*^ | < 0.05 | < 0.05 | 0.15 |
| **Total MUFA (g/day)** ^†^ | T1 (<14.28) | 1.00 (Ref) | 1.00 (Ref) | 1.00 (Ref) |
|  | T2 (14.28 to < 16.98) | 1.21 (0.88-1.68) | 1.23 (0.89-1.72) | 1.13 (0.80-1.60) |
|  | T3 (≥ 16.98) | 1.26 (0.91-1.74) | 1.26 (0.91-1.76) | 1.06 (0.71-1.59) |
|  | Continuous (per SD increment) | 1.12 (0.98-1.28) | 1.13 (0.99-1.30) | 1.06 (0.88-1.27) |
|  | *P* for trend ^*^ | 0.17 | 0.17 | 0.77 |
| **Animal-based MUFA (g/day)** ^†^ | T1 (< 8.14) | 1.00 (Ref) | 1.00 (Ref) | 1.00 (Ref) |
|  | T2 (8.14 to < 10.71) | 0.95 (0.69-1.31) | 0.94 (0.68-1.30) | 0.91 (0.65-1.28) |
|  | T3 (≥ 10.71) | 1.61 (1.15-2.26) | 1.58 (1.13-2.23) | 1.52 (1.01-2.29) |
|  | Continuous (per SD increment) | 1.19 (1.04-1.36) | 1.18 (1.03-1.36) | 1.16 (0.98-1.39) |
|  | *P* for trend ^*^ | < 0.05 | < 0.05 | < 0.05 |
| **Plant-based MUFA (g/day)** ^†^ | T1 (< 5.12) | 1.00 (Ref) | 1.00 (Ref) | 1.00 (Ref) |
|  | T2 (5.12 to < 6.67) | 0.95 (0.67-1.33) | 0.99 (0.70-1.40) | 1.00 (0.71-1.41) |
|  | T3 (≥ 6.67) | 0.83 (0.60-1.15) | 0.88 (0.63-1.23) | 0.83 (0.59-1.17) |
|  | Continuous (per SD increment) | 0.93 (0.82-1.07) | 0.95 (0.83-1.09) | 0.92 (0.80-1.06) |
|  | *P* for trend ^*^ | 0.26 | 0.45 | 0.27 |
| **Total PUFA (g/day)** ^†^ | T1 (< 6.15) | 1.00 (Ref) | 1.00 (Ref) | 1.00 (Ref) |
|  | T2 (6.15 to < 7.61) | 1.21 (0.86-1.70) | 1.24 (0.88-1.76) | 1.17 (0.83-1.68) |
|  | T3 (≥ 7.61) | 1.03 (0.75-1.41) | 1.06 (0.76-1.46) | 0.90 (0.61-1.33) |
|  | Continuous (per SD increment) | 0.97 (0.85-1.11) | 0.99 (0.86-1.13) | 0.88 (0.74-1.06) |
|  | *P* for trend ^*^ | 0.98 | 0.85 | 0.52 |
| **Total omega-3 PUFA ^c^ (g/day)** ^†^ | T1 (< 0.72) | 1.00 (Ref) | 1.00 (Ref) | 1.00 (Ref) |
|  | T2 (0.72 to < 0.90) | 1.29 (0.92-1.81) | 1.27 (0.91-1.79) | 1.22 (0.85-1.74) |
|  | T3 (≥ 0.90) | 1.07 (0.78-1.47) | 1.10 (0.79-1.52) | 0.96 (0.64-1.46) |
|  | Continuous (per SD increment) | 1.03 (0.90-1.18) | 1.04 (0.91-1.19) | 0.96 (0.78-1.18) |
|  | *P* for trend ^*^ | 0.77 | 0.65 | 0.78 |
| **Total omega-6 PUFA ^d^ (g/day)** ^†^ | T1 (< 5.43) | 1.00 (Ref) | 1.00 (Ref) | 1.00 (Ref) |
|  | T2 (5.43 to < 6.70) | 1.16 (0.82-1.63) | 1.22 (0.86-1.73) | 1.16 (0.81-1.66) |
|  | T3 (≥ 6.70) | 0.97 (0.70-1.33) | 0.99 (0.72-1.38) | 0.84 (0.57-1.23) |
|  | Continuous (per SD increment) | 0.96 (0.84-1.10) | 0.97 (0.85-1.11) | 0.88 (0.74-1.04) |
|  | *P* for trend ^*^ | 0.74 | 0.84 | 0.31 |
| **Marine omega-3 PUFA ^e^ (g/day)** ^†^ | T1 (< 0.04) | 1.00 (Ref) | 1.00 (Ref) | 1.00 (Ref) |
|  | T2 (0.04 to < 0.09) | 0.97 (0.70-1.34) | 0.92 (0.66-1.27) | 0.88 (0.63-1.24) |
|  | T3 (≥ 0.09) | 1.07 (0.77-1.49) | 1.00 (0.71-1.39) | 0.96 (0.68-1.35) |
|  | Continuous (per SD increment) | 1.06 (0.93-1.22) | 1.04 (0.91-1.19) | 1.02 (0.88-1.18) |
|  | *P* for trend ^*^ | 0.63 | 0.94 | 0.90 |
| **Omega-6/Omega-3 ratio** ^†^ | T1 (< 6.90) | 1.00 (Ref) | 1.00 (Ref) | 1.00 (Ref) |
|  | T2 (6.90 to < 7.93) | 1.13 (0.81-1.57) | 1.15 (0.82-1.61) | 1.16 (0.83-1.63) |
|  | T3 (≥ 7.93) | 0.83 (0.60-1.15) | 0.86 (0.62-1.20) | 0.87 (0.61-1.22) |
|  | Continuous (per SD increment) | 0.93 (0.81-1.06) | 0.94 (0.82-1.08) | 0.94 (0.81-1.08) |
|  | *P* for trend ^*^ | 0.19 | 0.28 | 0.33 |

Abbreviations: BMI, body mass index; CI, confidence interval; FA, fatty acid; MUFA, monounsaturated fatty acid; OR, odds ratio; PUFA, polyunsaturated fatty acid; Ref, reference; SD, standard deviation; SFA, saturated fatty acid; T, tertile.

The standard deviation for the listed fat and fatty acid are 8.69 g/day, 8.16 g/day, 6.88 g/day, 8.07 g/day, 3.60 g/day, 0.41 g/day, 3.54 g/day, 3.59 g/day, 3.34 g/day, 2.48 g/day, 2.21 g/day, 0.29 g/day, 1.97 g/day, 0.06 g/day, and 1.63 respectively.

Model 1: adjusted for age (continuous, years) and total energy intake (continuous, kcal/day).

Model 2: same as model 1 and further adjusted for BMI (continuous, kg/m^2^), alcohol drinking (yes or no), cigarette smoking (yes or no), household income (< 50, 50 to <100, or ≥ 100 RMB, thousand yuan), education (junior secondary or below, senior high school/technical secondary school, and junior college/university or above), physical activity (continuous, MET/hours/week), and abstinence time (continuous, days).

Model 3: same as model 2 and further adjusted for total protein (continuous, g/day) and total carbohydrate (continuous, g/day) intake.

^†^ Energy adjustment by residual method.

^*^ Test for trend based on variables containing the median value for each tertile.

^a^ Short-to-medium-chain SFA included saturated butyric (C4), caproic (C6), caprylic (C8), capric (C10), undecanoic (C11), lauric (C12), tridecanoic (C13) acids.

^b^ Long-chain SFA included saturated myristic (C14), pentadecanoic (C15), palmitic (C16), heptadecanoic (C17), stearic (C18), nonadecanoic (C19), arachidic (C20), behenic (C22), lignoceric (C24) acids.

^c^ Total omega-3 PUFA included alpha-linolenic acid, parinaric acid, docosatrienoic acid, eicosapentaenoic acid (EPA), docosapentaenoic acid (DPA), and docosahexaenoic acid (DHA).

^d^ Total omega-6 PUFA included linoleic acid, eicosadienoic acid, arachidonic acid, and docosatetraenoic acid.

^e^ Marine omega-3 PUFA included eicosapentaenoic acid (EPA), docosapentaenoic acid (DPA), and docosahexaenoic acid (DHA).
